# Conserved Pbp1/Ataxin-2 regulates retrotransposon activity and connects polyglutamine expansion-driven protein aggregation to lifespan-controlling rDNA repeats

**DOI:** 10.1038/s42003-018-0187-3

**Published:** 2018-11-05

**Authors:** Lauren A. Ostrowski, Amanda C. Hall, Kirk J. Szafranski, Roxanne Oshidari, Karan J. Abraham, Janet N. Y. Chan, Christian Krustev, Kevin Zhang, Ashley Wang, Yupeng Liu, Ru Guo, Karim Mekhail

**Affiliations:** 1 Department of Laboratory Medicine and Pathobiology, Faculty of Medicine, University of Toronto, Toronto, Ontario M5G 1M1 Canada; 2 Canada Research Chairs Program, Faculty of Medicine, University of Toronto, Toronto, Ontario M5G 1M1 Canada

## Abstract

Ribosomal DNA (rDNA) repeat instability and protein aggregation are thought to be two major and independent drivers of cellular aging. Pbp1, the yeast ortholog of human ATXN2, maintains rDNA repeat stability and lifespan via suppression of RNA–DNA hybrids. ATXN2 polyglutamine expansion drives neurodegeneration causing spinocerebellar ataxia type 2 and promoting amyotrophic lateral sclerosis. Here, molecular characterization of Pbp1 revealed that its knockout or subjection to disease-modeling polyQ expansion represses Ty1 (Transposons of Yeast) retrotransposons by respectively promoting Trf4-depedendent RNA turnover and Ty1 Gag protein aggregation. This aggregation, but not its impact on retrotransposition, compromises rDNA repeat stability and shortens lifespan by hyper-activating Trf4-dependent turnover of intergenic ncRNA within the repeats. We uncover a function for the conserved Pbp1/ATXN2 proteins in the promotion of retrotransposition, create and describe powerful yeast genetic models of ATXN2-linked neurodegenerative diseases, and connect the major aging mechanisms of rDNA instability and protein aggregation.

## Abstract (web summary)

Lauren Ostrowski, Amanda Hall et al. report a molecular characterization of Pbp1, the yeast ortholog of the neurodegenerative disease-associated human ATXN2. They find that *Pbp1* knockout or polyQ expansion compromises rDNA repeat stability via a mechanism linking neurodegenerative protein aggregation and aging.

## Introduction

Repetitive DNA sequences, which make up over half of the genome in many organisms, are critical to genome function^1,2^. However, due to their susceptibility to aberrant recombination and mobilization within genomes, repetitive DNA loci also constitute a major threat to genome integrity^3,4^. For instance, DNA recombination events within tandem or interspersed repetitive DNA sequences can give rise to lifespan-shortening chromosomal rearrangements^5–8^. It is therefore vital that cells tightly regulate repetitive DNA loci. This is especially applicable to transposable elements and ribosomal DNA (rDNA) repeats, which together constitute the majority of repetitive DNA sequences in eukaryotes^9^.

Transposons are genetic elements that move within the genome^10^. They comprise two classes reflecting the different mechanisms by which they transpose. Retrotransposons move via a copy-and-paste mechanism involving an RNA intermediate, while DNA transposons move through cut-and-paste processes^11^. In the budding yeast *Saccharomyces cerevisiae*, endogenous transposons are termed transposons of yeast (Ty)^12,13^. Ty elements are retrotransposons with flanking long terminal repeats (LTR) and are categorized into five families, of which the Ty1 family is the most abundant and active^12,13^. Ty1 is transcribed by RNA polymerase II (RNA Pol II) and comprises *TYA* and *TYB* open reading frames that respectively encode for the retrotransposition-mediating nucleocapsid-like protein Gag and the enzymatic Pol proteins protease, integrase and reverse-transcriptase^12^. Upon formation and maturation of a virus-like particle, Ty1 mRNA is reverse-transcribed into double-stranded cDNA that associates with integrase and integrates within new loci, including hotspots upstream of RNA Pol III-transcribed genes. Like other retroelements such as human LINE-1 (long interspersed nuclear element 1), Ty1 copy number increases with each round of retrotransposition and Ty1 is therefore regulated to ensure genome integrity^12,14–19^. In fact, retrotransposon dysregulation is a feature of age-related human diseases, including cancer and neurodegenerative disorders^20,21^.

Like transposons, rDNA repeats are highly controlled, as their dysregulation disrupts genome integrity^22,23^. In *S. cerevisiae*, ~190 rDNA units are tandemly arranged on chromosome XII (Chr. XII)^22,24^. Each unit is composed of a ribosomal RNA (rRNA) gene and an intergenic spacer (IGS; composed of IGS1 and IGS2). Within IGS1, binding of the replication fork block 1 (Fob1) protein stalls replication forks, thereby increasing the risk of unequal or aberrant recombination within the repeats^25,26^. Moreover, excessive transcription of non-coding RNA (ncRNA) species by RNA Pol II across the IGS can displace chromosomal cohesin complexes, thereby promoting aberrant rDNA recombination^27^. Potentially deleterious effects of excessive IGS ncRNA levels are abated by Sir2 histone deacetylase-mediated chromatin silencing and Trf4 poly(A) polymerase-dependent ncRNA turnover^28–33^. Still, ncRNAs can engage in RNA–DNA hybrids (R-loops), causing replication fork stalling and rDNA instability^34^. However, interaction of ncRNAs with the conserved RNA-binding protein Pbp1 (Pab1-binding protein 1) prevents R-loop buildup^34,35^.

The human ortholog of yeast *PBP1* is the *ATXN2* gene^36^. Similar to Pbp1 deletion, repression of the ATXN2 protein leads to R-loop accumulation and rDNA/genome instability in human cells^35^. Importantly, *ATXN2* mutations are associated with neurodegenerative diseases. Specifically, the N-terminal region of the ATXN2 protein contains a polyglutamine (polyQ) tract constituted of ~23 glutamines (Qs). Expansion of this tract to 27–33 Qs promotes amyotrophic lateral sclerosis (ALS; a.k.a. Lou Gehrig’s Disease) and/or spinocerebellar ataxia type 2 (SCA2) while expansions beyond 33 Qs are the genetic cause of SCA2 disease^37–40^.

The stability of rDNA repeats is critical to replicative lifespan, which is defined by the number of times that a yeast mother cell replicates DNA and yields progeny before reaching senescence^41,42^. Aberrant rDNA repeat recombination and/or its extrachromosomal circle by-products have been proposed to shorten cellular lifespan^5–7,43^. Consistent with the centrality of rDNA to lifespan, deletion of Fob1, which is required for recombination within rDNA repeats, hyper-stabilizes the repeats and extends lifespan^44,45^. In addition to rDNA instability, the accumulation of protein aggregates has been proposed to be another major mechanism of cellular aging^46–48^. But how protein aggregates shorten lifespan is unclear.

Here we first find that Pbp1 sustains Ty1 retromobility by inhibiting Trf4-dependent turnover of Ty1 mRNA. We then create yeast genetic models of ATXN2 polyQ expansion diseases and use them to uncover that Pbp1 polyQ expansion inhibits Ty1 retromobility. This is due to an ability of polyQ-expanded proteins to induce aggregation of the Ty1 Gag protein, a process that we herein term *trans*-aggregation. Gag aggregates accumulate in the rDNA-harboring nucleolus and promote Trf4-dependent turnover of long intergenic ncRNAs within the rDNA IGS, leading to rDNA repeat instability and lifespan shortening without involving R-loop accumulation. Like our yeast models, ATXN2 loss and polyQ expansion differentially alter the expression of abundant human retroelements and rDNA IGS ncRNAs. Overall, we ascribe a function to Pbp1/ATXN2 in transposon control, create yeast genetic models of disease-linked ATXN2 polyQs, and connect the rDNA instability and protein aggregation mechanisms of aging.

## Results

### Pbp1 promotes Ty1 retrotransposition

**Fig. 1:**
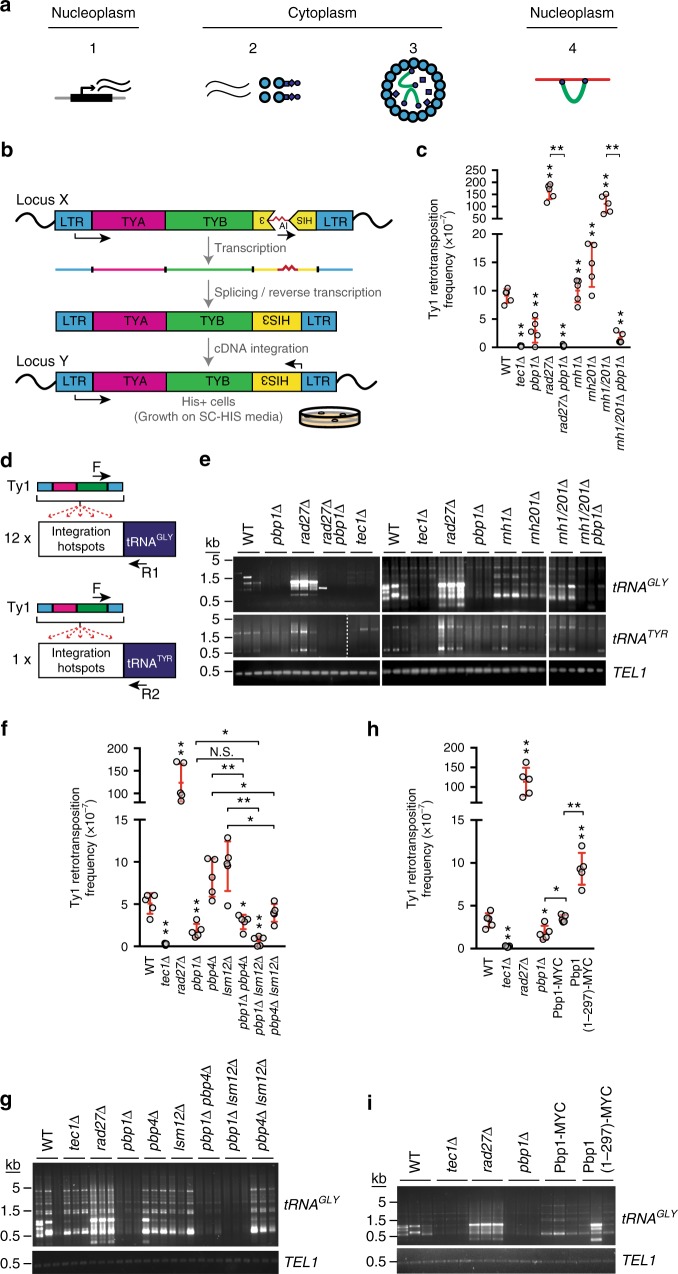
The rDNA-stabilizing Pbp1 paradoxically promotes Ty1 retrotransposition. **a** Transcription of a Ty1 element/gene (1), translation (2), cDNA synthesis inside virus-like particles (3), and hotspot integration (4). Larger circles = Ty1 Gag protein; Small circles/squares = Ty1 protease, integrase, and reverse-transcriptase; Horseshoe = Ty1 cDNA. **b** Schematic illustrating *Ty1his3AI* retromobility assay. His^−^ cells become His^+^ only after a complete retrotransposition event has occurred with splicing of the artificial intron. **c** Effect of *pbp1∆* on retromobility of *Ty1his3AI*. **d** Schematic illustrates Ty1 integration PCR assay. Ty1 cDNA integrates upstream of RNA Pol III-transcribed genes. PCR primers are indicated with arrows. **e** Semi-quantitative PCR products reflecting Ty1 integration upstream of 12 *tRNA*^*GLY*^ loci and one *tRNA*^*TYR*^ gene. **f** Effects of *lsm12∆* and *pbp4∆* on retromobility of *Ty1his3AI*. **g** Semi-quantitative PCR products reflecting Ty1 integration upstream of 12 *tRNA*^*GLY*^ loci. **h** Frequency of *Ty1his3AI* retromobility in a Pbp1 truncation mutant lacking the C-terminal Pab-1-binding domain (also see Supplementary Fig. 1). **i** Semi-quantitative PCR products reflecting Ty1 integration upstream of 12 *tRNA*^*GLY*^ loci. **c**, **f**, **h** Mean ± SD; *n* = 5 independent cultures; Mann–Whitney *U*-test. **e**, **g**, **i** *TEL1* amplification served as control. **a**–**i** *p < 0.05; **p < 0.01

Given the role of Pbp1 in the regulation of rDNA repeats^34,35^, we asked whether Pbp1 also regulates the repetitive retrotransposons. Gag is a key component of the Ty1 retrotransposition lifecycle (Fig. 1a). To test whether Pbp1 impacts Ty1 retrotransposition, we employed two experimental systems. First, we used the *Ty1his3AI* reporter system, which measures the frequency of retromobility of a single Ty1 reporter gene across the genome (Fig. 1b)^49^. In this reporter, an inverted *HIS3* gene harboring a forward artificial intron is integrated in the genome of *HIS*-negative cells. Transcription of the full reporter is followed by artificial intron splicing (occurs ~3% of the time) and reverse-transcription of the spliced mRNA into double-stranded cDNA, which is then integrated into the genome allowing for *HIS3* gene expression (Fig. 1b)^49^. Using the *Ty1his3AI* reporter system, we confirmed that deletion of the Ty1 transcription factor Tec1 (transposon enhancement control 1) decreases retrotransposition (Fig. 1c). We also confirmed that loss of the Ty1 cDNA repressor Rad27 (radiation sensitive 27) or the Ty1 RNA-cDNA hybrid-repressing RNaseH enzymes (Rnh1 and Rnh201) greatly induce retrotransposition (Fig. 1c), as expected^50–52^. As Pbp1 is known to limit recombinational instability at rDNA, we expected Pbp1 to repress retromobility. In contrast, *PBP1* knockout (*pbp1Δ*) decreased retromobility in wild type, *rad27Δ*, and *rnh1/201Δ* cells (Fig. 1c). We also assessed if Pbp1 impacts endogenous *Ty1* activity at retrotransposition hotspots upstream of tRNA genes for glycine and tyrosine (*tRNA*^*GLY*^ and *tRNA*^*TYR*^) (Fig. 1d). This allows for the assessment of Ty1 mobility at dozens of endogenous integration hotspots. We observed that *pbp1Δ* greatly decreased endogenous retrotransposition in wild type, *rad27Δ*, and *rnh1/201Δ* cells (Fig. 1e). These data reveal that Pbp1 sustains retrotransposition both in wild-type cells and in mutants with elevated retromobility.

We then set out to assess the potential role of the Pbp1-Pbp4-Lsm12 complex and its binding partner Pab1 in retrotransposition. Using the *Ty1his3AI* reporter, we found that neither *pbp4Δ* nor *lsm12Δ* altered Ty1 retromobility (Fig. 1f). However, deleting Lsm12, but not Pbp4, in *pbp1Δ* cells further decreased retromobility (Fig. 1f). Combining *pbp4Δ* and *lsm12Δ* was ineffectual on *Ty1his3AI* reporter retromobility (Fig. 1f). Similar observations were made when we examined the impact of single and double deletions of *PBP1*, *PBP4*, and *LSM12* on Ty1 integration at retrotransposition hotspots upstream of *tRNA*^*GLY*^ genes (Fig. 1g). The Pbp1-Pbp4-Lsm12 complex physically interacts with Pab1. However, replacing endogenous Pbp1 with a Pbp1 mutant that harbors the Like-Sm (Lsm) and Lsm-AD (Lsm-Associated) RNA-binding domains, but not the Pab1-binding domain, did not phenocopy the repressive impact of *pbp1Δ* on *Ty1his3AI* or endogenous *Ty1-tRNA*^*GLY*^ reporter retromobility (Fig. 1h, i; Supplementary Fig. 1). In fact, loss-of-Pab1 binding moderately, yet significantly, increased retromobility (Fig. 1h–i, Mann–Whitney *U*-test *p*-value <0.05). This indicates that the loss of Pbp1 compromises retrotransposition independently of Pab1. Thus, our data so far reveal that Lsm12 and especially the Pbp1 subunit of the Pbp1-Pbp4-Lsm12 complex can promote Ty1 retromobility.

### Pbp1 represses Trf4-dependent Ty1 mRNA turnover

**Fig. 2:**
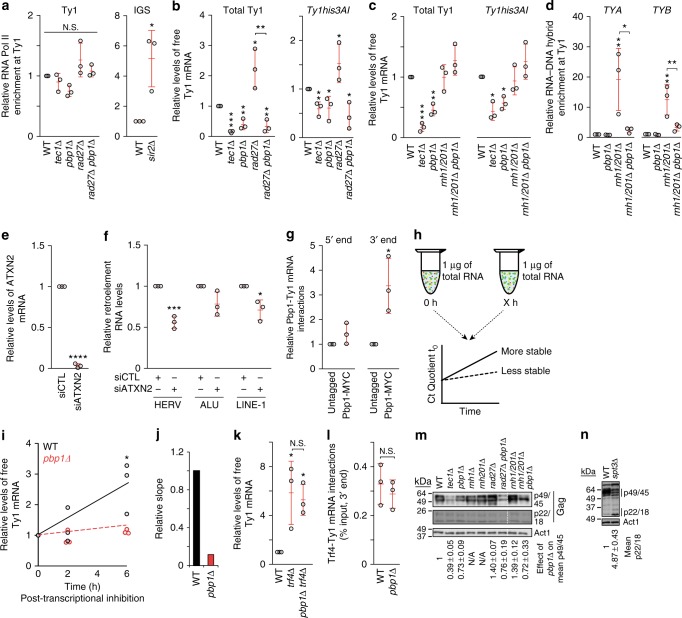
Pbp1 represses Trf4-dependent Ty1 mRNA turnover. **a** ChIP examining the localization of RNA Pol II at Ty1 using an α-RNA Pol II (phosphorylated serine 5) antibody. (One-way ANOVA and Dunnett’s post hoc for left panel, Student’s *t*-test for right panel). **b**, **c** Effects of *pbp1∆* on the levels of total Ty1 and *Ty1his3AI* mRNA as determined by RT-qPCR (Student’s *t*-test). **d** ChIP examining RNA–DNA hybrid levels at Ty1 using the S9.6 antibody (One-way ANOVA and Sidak’s post hoc). **e** Confirmation of ATXN2 knockdown by RT-qPCR in siRNA-transfected HEK293T cells. Values are normalized to GAPDH (Student’s *t*-test). **f** Effect of ATXN2 knockdown on levels of retroelement RNAs detected by RT-qPCR. Values are normalized to GAPDH and statistics are relative to siRNA control (siCTL; Student’s *t*-test). **g** Interactions between Pbp1-MYC and Ty1 mRNA. Presented are levels of Ty1 mRNA, immunoprecipitated using an α-MYC antibody, relative to input as detected by RT-qPCR (Student’s *t*-test). **h** Schematic illustrating RNA stability assay. Transcription is chemically inhibited, and relative abundance of Ty1 mRNA in 1 μg of total RNA is quantified at various time-points by RT-qPCR. More stable RNA displays a greater slope. **i** Stability of Ty1 mRNA as assessed with RT-qPCR at several time-points following RNA Pol II inhibition. Line of best fit is plotted (Student’s *t*-test). **j** Quantification of slopes in **i**. **k** Effect of *pbp1∆* on the level of Ty1 mRNA in the absence of TRF4 as detected by RT-qPCR (One-way ANOVA and Tukey’s post hoc). **l** Interactions between Trf4 and Ty1 mRNA. Presented are levels of Ty1 mRNA co-immunoprecipitating with Trf4 relative to input as detected by RT-qPCR. Values are normalized to mock pull-down from *trf4∆* cells (Student’s *t*-test). **m**, **n** Western blot examining the levels (*n* = 2) of Ty1 Gag forms p49 (unprocessed), p45 (processed) and p22/18 (truncated). In control *spt3*∆ cells, p22/18 levels are induced while p49/45 levels are decreased. **a**–**g**, **i**, **k**, **l** Mean ± SD; *n* *=* 3. **a**–**d**, **g** Statistics are relative to levels in wild-type cells. **a**–**n** *p < 0.05; **p < 0.01; ***p < 0.001; ****p < 0.0001

To uncover how Ty1 retromobility is repressed upon Pbp1 loss, we assessed the impact of that loss on different Ty1 lifecycle stages (Fig. 1a). Chromatin immunoprecipitation (ChIP) revealed that *pbp1Δ* does not alter the enrichment of RNA Pol II at Ty1 (Fig. 2a). However, the loss of Pbp1 from wild type and *rad27∆* cells decreased total Ty1 mRNA and *Ty1his3AI* reporter mRNA levels (Fig. 2b). In contrast, the loss of Pbp1 from *rnh1/201Δ* cells did not alter Ty1 mRNA or *Ty1his3AI* mRNA levels (Fig. 2c). As the increased retromobility observed in *rnh1/201Δ* cells is driven by Ty1 RNA-cDNA hybrid accumulations and not changes in free RNA levels^51,52^, the effect of Pbp1 on Ty1 mRNA in this strain may be masked by the fact that the RNA is trapped in RNA–DNA hybrids. Indeed, ChIP revealed that the loss of Pbp1 decreased Ty1 RNA-cDNA hybrid levels in *rnh1/201Δ* cells but had no effect in wild-type cells (Fig. 2d). To test whether the retroelement-promoting role of Pbp1 is conserved, we performed siRNA-mediated knockdown of the Pbp1 ortholog ATXN2 in human HEK293T cells (Fig. 2e). We then measured RNA levels of the retroelements HERV (human endogenous retrovirus), Alu (Arthrobacter luteus elements), and LINE-1. Knockdown of human ATXN2 decreased the levels of HERV and LINE-1 transcripts, suggesting a conserved role for Ataxin-2 proteins in the promotion of retroelement expression (Fig. 2e, f). Next, RNA immunoprecipitation (RIP) revealed that Pbp1 directly or indirectly interacts with the 3′-end of Ty1 mRNA (Fig. 2g). In fact, Pbp1 loss compromised Ty1 mRNA half-life in mRNA stability assays (Fig. 2h–j). The 3′-end of Ty1 mRNA is typically recognized and targeted for degradation by the Trf4 polyadenylase^16^. Thus, we asked if Pbp1 may be limiting Trf4-dependent turnover of Ty1 mRNA. Consistent with this possibility, we confirmed that Pbp1 promotes while Trf4 limits Ty1 mRNA levels (Fig. 2, compare k to b-c). More importantly, Pbp1 loss failed to lower Ty1 mRNA levels in *trf4Δ* cells (Fig. 2k), suggesting that Pbp1 restricts Trf4 activity or binding at Ty1 mRNA. However, Pbp1 loss did not affect Trf4 binding to Ty1 mRNA, suggesting that Pbp1 rather limits Trf4 activity at Ty1 mRNA (Fig. 2l). Consistent with the general ability of Pbp1 to promote Ty1 mRNA stability, Pbp1 also promoted Ty1 full-length Gag protein levels (p49/45), but not truncated Gag protein levels (p22/18), in wild type, *rad27Δ*, and *rnh1/201Δ* cells (Fig. 2m, n; Supplementary Fig. 2). Taken together, our results reveal that Pbp1 binds to the 3′-end of Ty1 mRNA where it restricts the ability of Trf4 to mediate the turnover of Ty1 mRNA.

### Humanization of yeast Pbp1 with disease-linked polyQs

**Fig. 3:**
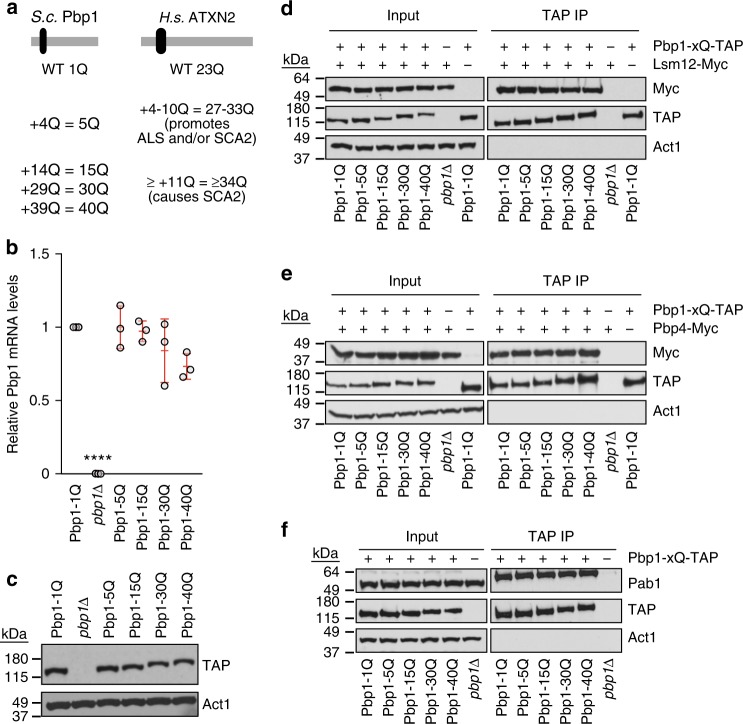
Humanization of yeast Pbp1 with neurodegeneration-linked polyQ expansion. **a** Schematic of Pbp1-polyQ expansions generated in yeast to reflect ALS/SCA2 human genetics. **b** Effects of Pbp1-polyQ expansion on the level of Pbp1 transcripts. Shown are Pbp1 mRNA levels as detected by RT-qPCR (Mean ± SD; *n* *=* 3). Values and statistics are relative to levels in Pbp1-1Q cells (one-way ANOVA and Dunnett’s post hoc; ****p < 0.0001). **c** Effects of Pbp1-polyQ expansions on Pbp1 protein levels. Levels of Pbp1-TAP were detected by western blotting. **d**–**f** CoIP examining interactions between Pbp1-polyQ and Lsm12 **d**, Pbp4 **e**, and Pab1 **f**

The human ortholog of yeast Pbp1 is ATXN2, which exhibits moderate or large expansions of its polyQ tract in the neurodegenerative diseases ALS and SCA2, respectively^36^. Yeast models of Huntington’s disease and Parkinson’s disease have played critical roles in uncovering determinants of the deleterious effects of the huntington and α-synuclein proteins^53,54^. However, these genetic models consisted of expressing, in yeast, exogenous human factors that do not have a natural yeast ortholog. This made it impossible to employ the power of yeast proteomics and genetics to compare how the loss or polyQ expansion of an evolutionarily conserved neurodegeneration-linked protein differentially impacts conserved molecular processes and phenotypes. Thus, we created yeast genetic models carrying a *PBP1* gene encoding different human disease-associated polyQ expansions (Fig. 3a). The models consisted of cells carrying Pbp1 that is either wild type (1Q), containing a 4Q expansion similar to ALS/SCA2-promoting ATXN2 or harboring a 14Q, 29Q, or 39Q expansion similar to SCA2-causing ATXN2 (Fig. 3a). This results in genetic models in which Pbp1 has 1Q, 5Q, 15Q, 30Q, or 40Q tracts (Fig. 3a). Importantly, all of these different Pbp1 forms were expressed to the same level (Fig. 3b, c). Unexpectedly, Pbp1 polyQ expansion did not impact the physical interaction of Pbp1 with its binding partners Lsm12, Pbp4, or Pab1 (Fig. 3d–f). Thus, we have created multiple yeast Pbp1 genetic models of ATXN2 polyQ expansion in which no overt disruptions of major Pbp1 protein interactions are observed.

### Pbp1 polyQs inhibit Ty1 at the post-translational level

**Fig. 4:**
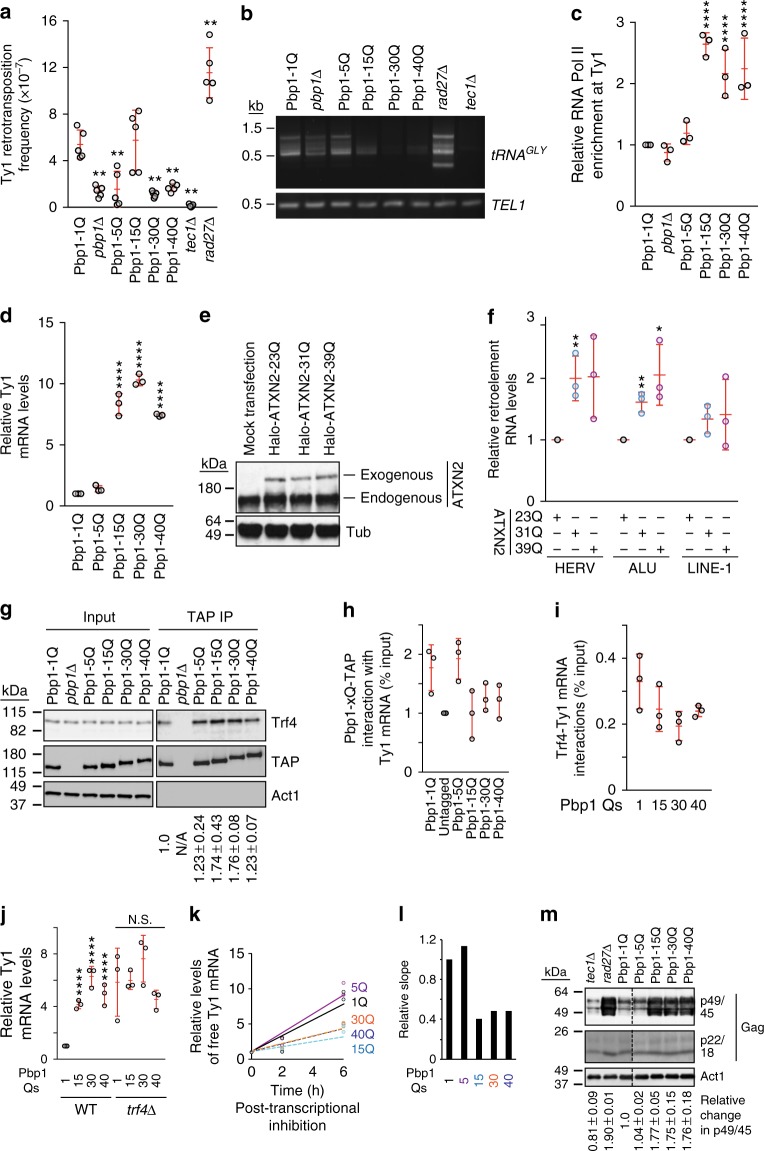
Pbp1 polyQ expansions inhibit Ty1 retromobility at the post-translational level. **a** Effects of Pbp1-polyQ expansion on *Ty1his3AI* retromobility (Mean ± SD; *n* *=* 5 independent cultures; Mann–Whitney *U*-test). **b** Semi-quantitative PCR products reflecting Ty1 integration upstream of 12 *tRNA*^*GLY*^ loci. *TEL1* amplification serves as control. **c** ChIP examining the localization of RNA Pol II at *TyB* using an α-RNA Pol II (phosphorylated serine 5) antibody (One-way ANOVA and Dunnett’s post hoc). **d** Effects of Pbp1-polyQ expansion on the levels of Ty1 mRNA in RT-qPCR (One-way ANOVA and Dunnett’s post hoc). **e** Confirmation of ATXN2-polyQ expression in transfected HEK293T cells by western blotting. **f** Effects of ATXN2 polyQ expansion on the levels of retroelement RNAs as detected by RT-qPCR. Values are normalized to GAPDH and statistics are relative to 23Q (Student’s *t*-test). **g** Pbp1 polyQ forms exhibit an increased interaction with Trf4 (*n* = 2). TAP-tagged Pbp1 proteins with indicated glutamine features were subjected to anti-TAP pulldowns followed by immunoblotting for endogenous Trf4, TAP tag or Actin control. **h**, **i** Interactions between Pbp1 forms or Trf4 with Ty1 mRNA. Presented are levels of Ty1 mRNA immunoprecipitated relative to input, as detected by RT-qPCR. Values are normalized to pull-down in untagged (**h**) or *trf4∆* cells (**i**). **j** Effects of Pbp1-polyQ expansion on Ty1 mRNA levels in the presence/absence of TRF4 as detected by RT-qPCR (One-way ANOVA and Dunnett’s post hoc). **k** Ty1 mRNA stability assay in Pbp1 polyQ cells. Following RNA Pol II inhibition, Ty1 mRNA levels were assessed over time by RT-qPCR and the line of best fit was plotted (*n* = 2). **l** Quantification of slopes in **k**. **m** Western blot examining in Pbp1-polyQ-expanded cells (*n* = 2), the levels of Ty1 Gag forms p49 (unprocessed), p45 (processed), and p22/18 (truncated). **c**, **d, f**, **h**–**j** Mean ± SD; *n* = 3. **c**, **d**, **j** Statistics are presented relative to levels detected in Pbp1-1Q cells. **a**–**m** *p < 0.05; **p < 0.01; ****p < 0.0001

Next, to assess whether Pbp1 polyQs behave similar to Pbp1 deletion in terms of disrupting Ty1 retromobility at the RNA turnover level, we monitored both *Ty1his3AI* reporter mobility and endogenous Ty1 activity upstream of *tRNA*^*GLY*^ integration hotspots. Using both approaches, Pbp1 30Q and 40Q mimicked *pbp1Δ* and decreased *Ty1his3AI* reporter retromobility (Fig. 4a, b). Pbp1 5Q decreased only *Ty1his3AI* reporter mobility but not global endogenous integration, while 15Q consistently compromised endogenous retrotransposition but not *Ty1his3AI* reporter mobility (Fig. 4a, b). The *Ty1his3AI* reporter measures the ability of one retroelement to move once while endogenous retromobility upstream of *tRNA*^*GLY*^ can reflect numerous Ty1 elements moving multiple times. Thus, giving more weight to the results of endogenous data, when comparing the impact of *pbp1Δ* to Pbp1 polyQs on endogenous retromobility upstream of *tRNA*^*GLY*^, we conclude that Pbp1 15Q, 30Q, and 40Q exhibit lower retromobility than wild type and even *pbp1Δ* cells (Fig. 4b). Taken together, these data indicate that Pbp1 15Q and especially 30Q and 40Q exert a repressive gain of function or mimic Pbp1 loss of function at Ty1 retroelements.

Thus, we assessed the impact of Pbp1 polyQs across the Ty1 lifecycle. Unexpectedly, we observed changes that are consistent with Ty1 activation, not inhibition. First, we observed that Pbp1 15Q, 30Q, and 40Q, but not 5Q or *pbp1Δ*, exhibited increased RNA Pol II levels at Ty1, as well as elevated Ty1 mRNA levels (Fig. 4c, d). To test whether similar effects are observed at human retroelements, we transfected human HEK293T cells with plasmids harboring either wild type (23Q), 31Q or 39Q versions of ATXN2 and measured the levels of HERV, Alu and LINE-1 transcripts (Fig. 4e, f)^55^. Similar to our yeast models, disease-linked polyQ expansion of ATXN2 increased HERV and/or Alu transcript levels (Fig. 4f). We next assessed the impact of Pbp1 polyQs on the pairwise interactions between Pbp1, Trf4, and Ty1 mRNA. All polyQ forms interacted more with Trf4, while Pbp1 15Q, 30Q, and 40Q lost the ability to interact with Ty1 mRNA (Fig. 4g, h). However, no significant changes were detected when we examined Trf4-Ty1 mRNA interactions (Fig. 4i). Nonetheless, Pbp1 15Q, 30Q, and 40Q failed to increase Ty1 mRNA levels in the absence of Trf4, suggesting that these Pbp1 polyQ forms increase the levels of Ty1 mRNA by primarily compromising Trf4 activity at Ty1 (Fig. 4j). Despite its accumulation in Pbp1 15Q, 30Q, and 40Q cells, Ty1 mRNA actually exhibited a shorter half-life, suggesting that Trf4-independent processes may be preventing further increases in the steady-state levels of Ty1 mRNA (Fig. 4k, l). Most importantly, Pbp1 15Q, 30Q, and 40Q cells exhibited greatly elevated Ty1 Gag protein levels (Fig. 4m). Although these increases are likely reflective of increased Ty1 mRNA levels in the mutants, we cannot completely rule out the possibility that these increases may be partially due to higher Gag protein stability. Thus, our findings reveal that the SCA2-modeling Pbp1 15Q, 30Q, and 40Q repress Ty1 retromobility despite promoting RNA Pol II localization to Ty1 loci and limiting Trf4 localization and function at Ty1 mRNA. These findings also suggest that Pbp1 15Q, 30Q, and 40Q exert a dominant retrotransposition-inhibiting effect at a step succeeding Gag protein translation.

### PolyQs block Ty1 via protein aggregation and promote aging

**Fig. 5:**
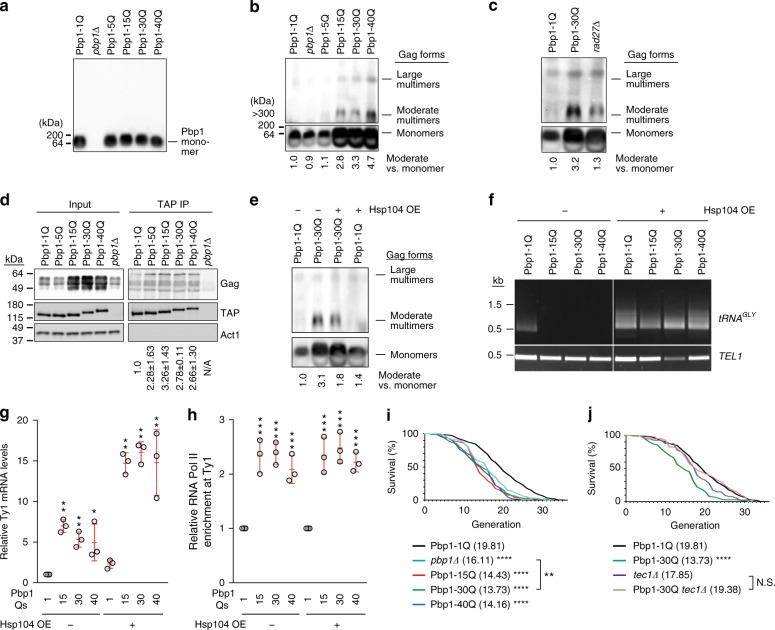
Pbp1 PolyQ expansions induce retrotransposon-inhibiting protein aggregation and shorten replicative lifespan. **a** SDD-AGE of Pbp1 variants. **b** SDD-AGE of Gag in Pbp1-polyQ cells. Shown below blots is multimer quantification relative to Pbp1-1Q cells. **c** SDD-AGE of Gag in *rad27*∆ cells. Shown below blots is multimer quantification relative to Pbp1-1Q cells. **d** CoIP examining interactions between Pbp1 variants and Ty1 Gag (*n* = 2). **e** SDD-AGE of Gag in Pbp1-polyQ-expanded cells in the absence/presence of Hsp104 over-expression. Shown below blots are multimer quantification relative to Pbp1-1Q cells. **f** Semi-quantitative PCR products reflecting Ty1 integration upstream of 12 *tRNA*^*GLY*^ loci in cells with Pbp1 variants and Hsp104 over-expression. *TEL1* amplification served as control. **g** Effects of Hsp104 over-expression on Ty1 mRNA levels in cells with Pbp1 variants. Relative mRNA levels shown were detected by RT-qPCR (Mean ± SD; *n* = 3). Statistics are relative to levels detected in Pbp1-1Q cells with/without Hsp104 over-expression (one-way ANOVA and Dunnett’s post hoc). **h** ChIP analysis examining the localization of RNA Pol II at Ty1 in cells with Pbp1 variants and Hsp104 over-expression. Presented are relative levels of Ty1 DNA co-immunoprecipitating with RNA Pol II (phosphorylated serine 5) relative to input, as detected by qPCR (Mean ± SD; *n* = 3). Statistics are relative to levels detected in Pbp1-1Q cells with/without Hsp104 over-expression (one-way ANOVA and Dunnett’s post hoc). **i** Effect of Pbp1-polyQ expansion on replicative lifespan. Values are plotted as a survival curve, and mean lifespans are indicated in parentheses. Statistics are relative to Pbp1-1Q (Mann–Whitney *U*-test). **j** Effect of decreased retromobility on replicative lifespan. Values are plotted as a survival curve, and mean lifespans are indicated in parentheses. Statistics are relative to Pbp1-1Q (Mann–Whitney *U*-test). **a**–**j** *p < 0.05; **p < 0.01; ***p < 0.001; ****p < 0.0001

One post-translational mechanism which could resolve the apparent discordance between increased Gag expression and decreased Ty1 retromobility is the formation of protein aggregates. Although we did not detect overt aggregation of wild type or polyQ forms of Pbp1 in semi-denaturing detergent agarose gel electrophoresis (SDD-AGE) (Fig. 5a), this method revealed that cells expressing Pbp1 15Q, 30Q, or 40Q, but not 5Q, exhibit increased levels of moderately sized Gag protein aggregates (Fig. 5b). Importantly, although *rad27Δ* cells exhibit similar levels of monomeric Gag protein expression compared to Pbp1 30Q cells, Gag aggregation is much lower in *rad27Δ* cells (Fig. 5c). This demonstrates that the aggregation of Gag is not simply promoted by its increased abundance. We also found that all polyQ-expanded Pbp1 proteins have a slightly elevated level of interaction with Gag (Fig. 5d). Our data so far suggest that SCA2-modeling Pbp1 proteins do not exhibit self-aggregation but instead operate in *trans* to induce the aggregation of Gag. We hereafter refer to this phenomenon as *trans*-aggregation. The ability of Pbp1-polyQ to *trans*-aggregate Gag may be direct, which may explain the increased interactions between Gag and Pbp1-polyQ proteins. Alternatively, *trans*-aggregation may be indirect.

Next, we asked whether over-expression of the protein disaggregase Hsp104 (heat shock protein 104)^56^ can decrease Gag aggregation and rescue Ty1 retromobility in cells with SCA2-linked Pbp1 polyQs. Indeed, Hsp104 over-expression decreased Gag aggregation in Pbp1 30Q cells and countered the inhibitory effect of Pbp1 15Q, 30Q, and 40Q onto Ty1 retromobility (Fig. 5e–f). Hsp104 over-expression did not decrease but further increased Ty1 mRNA levels in cells expressing Pbp1 15Q, 30Q, or 40Q (Fig. 5g). This may be due to the fact that Hsp104 over-expression does not abolish the elevated localization of RNA Pol II at Ty1 (Fig. 5h). Thus, in cells with SCA2-modeling Pbp1, Gag aggregation exerts a reversible inhibitory effect on retrotransposition.

As protein aggregation can compromise lifespan, we asked whether Pbp1 15Q, 30Q, or 40Q shorten replicative lifespan, which is the number of times that a yeast mother cell replicates DNA and yields progeny before senescence. We found that, similar to *pbp1Δ*, the Pbp1 15Q, 30Q, and 40Q forms all decreased lifespan (Fig. 5i and Supplementary Table 1). In fact, the lifespan of Pbp1 30Q cells was lower than the lifespan of *pbp1Δ* cells (Fig. 5i and Supplementary Table 1). To test whether the decreased lifespan observed under polyQ expansion conditions is due to the repression of Ty1 retromobility, we tested the impact of Pbp1 polyQ expansion in *tec1Δ* cells, which are deficient in Gag expression. We found that *tec1Δ* does not alter lifespan in wild-type cells (Fig. 5j and Supplementary Table 1). More importantly, Pbp1 30Q decreased the replicative lifespan of wild-type cells by 30%, but did not alter the lifespan of *tec1Δ* cells (Fig. 5j and Supplementary Table 1). These findings reveal that the inhibition of retromobility does not alter the replicative lifespan of wild-type cells. Instead, in SCA2-modeling cells, the Ty1 pathway contributes to lifespan shortening. This raises the possibility that Gag aggregation may shorten lifespan by exerting a deleterious effect elsewhere in the cell.

### R-loop/chromatin-independent rDNA destabilization by polyQs

**Fig. 6:**
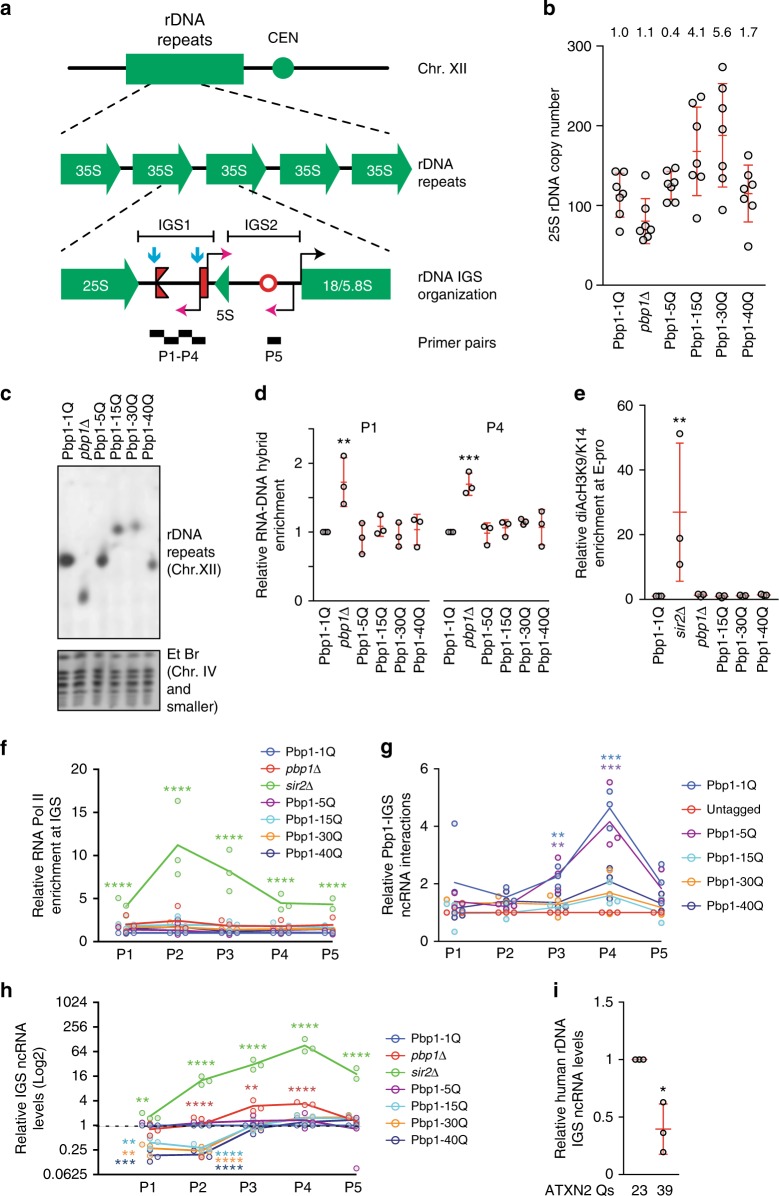
Pbp1 polyQ expansions destabilize rDNA repeats independently of R-loops and chromatin. **a** rDNA repeats on Chr. XII. Expression of rRNA genes (black arrowhead) and IGS ncRNAs (magenta arrowheads) is shown. Rightward red fork = replication fork block; red rectangle = E-pro promoter; red circle = replication origin; blue arrows = R-loop accumulation-prone sites. Primers amplifying IGS regions P1-P5 are shown. **b** rDNA copy number analysis of Pbp1-polyQ-expanded cells detected by qPCR (Mean ± SD; *n* = 7). Variance is presented relative to Pbp1-1Q. **c** CHEF coupled with southern blotting of Chr. XII in Pbp1-polyQ-expanded cells. Bands on EtBr-stained gel served as loading control. **d** ChIP examining RNA–DNA hybrid levels at the IGS using the S9.6 antibody (Mean ± SD; *n* = 3; one-way ANOVA and Dunnett’s post hoc). **e** ChIP examining the enrichment of diAcH3K9/K14 open chromatin marks relative to that of total H3 at the rDNA IGS (Mean ± SD; *n* = 3). Statistics are relative to levels detected in Pbp1-1Q cells (one-way ANOVA and Dunnett’s post hoc). **f** ChIP examining the enrichment of RNA Pol II (phosphorylated serine 5) across the rDNA IGS (Mean ± SD; *n* = 3). Statistics are relative to levels detected in Pbp1-1Q cells (one-way ANOVA and Dunnett’s post hoc). **g** Interactions between Pbp1 forms and IGS ncRNAs. Presented are levels of IGS ncRNAs immunoprecipitated relative to input, as detected by RT-qPCR (Mean ± SD; *n* *=* 3). Values are normalized to pull-down from untagged cells. Statistics are relative to levels detected in untagged cells (one-way ANOVA and Dunnett’s post hoc). P1-P5 primers pair locations are in **a**. **h** Effects of Pbp1-polyQ expansions on the levels of IGS ncRNAs as detected by RT-qPCR (Mean ± SD; *n* = 3; Log2 scale). Statistics are presented relative to levels detected in Pbp1-1Q cells (one-way ANOVA and Dunnett’s post hoc). **i** Effects of human ATXN2-polyQ expansion on the levels of ncRNAs detected by RT-qPCR at three IGS sites (Mean ± SD; Student’s *t*-test). **a**–**i** *p < 0.05; **p < 0.01; ***p < 0.001; ****p < 0.0001

Destabilization of rDNA repeats (Fig. 6a) constitutes a major mechanism of replicative aging. Thus, we asked if SCA2-linked Pbp1 polyQ expansions disrupt rDNA repeat stability. Clonal analysis revealed that *pbp1Δ* decreased average rDNA copy numbers, as expected (Fig. 6b)^34^. In contrast, Pbp1 15Q and 30Q increased rDNA copy numbers (Fig. 6b). Moreover, Pbp1 15Q, 30Q, and 40Q cells exhibited increased rDNA copy number variance (Fig. 6b). Contour clamped homogenous electric field electrophoresis (CHEF) analysis coupled to southern blotting can be used to reveal key features of Chr. XII, which harbors rDNA repeats^34,43^. CHEF showed that *pbp1Δ* as well as Pbp1 15Q, 30Q, and 40Q, but not 5Q, decreased Chr. XII band intensity, pointing to partially increased heterogeneity in the size of the rDNA array across the cell population (Fig. 6c). Moreover, the fainter Chr. XII bands in *pbp1Δ* cells as well as Pbp1 15Q, 30Q, or 40Q cells migrated higher or lower than the Chr. XII band in wild type or Pbp1 5Q cells, pointing to the presence of a cell subpopulation with relatively stable rDNA repeats that harbor aberrant copy numbers (Fig. 6c). Taken together, our findings indicate that *pbp1Δ*, Pbp1 15Q, 30Q, and 40Q, but not 5Q, induce rDNA repeat instability. Moreover, Pbp1 15Q, 30Q, and 40Q especially increase rDNA copy number variance.

We next asked whether the mechanism underlying rDNA repeat destabilization in Pbp1 polyQ expansion cells is similar to the RNA–DNA hybrid-dependent mechanism causing rDNA instability in *pbp1Δ* cells^34,35^. However, unlike *pbp1Δ*, Pbp1 polyQs failed to increase RNA–DNA hybrid levels (Fig. 6d; Supplementary Fig. 3). In addition to RNA–DNA hybrid suppression, rDNA repeat stability is also reliant on the assembly of silent chromatin by Sir2 to help repress RNA Pol II-dependent ncRNA expression from the E-pro promoter within the rDNA IGS^28,29,31,33^. In contrast to *sir2Δ*, Pbp1 loss or polyQ expansions failed to alter the enrichment of the open chromatin mark Histone H3 di-acetylated on lysines 9 and 14 (diAcH3K9/14) or change the levels of RNA Pol II within the IGS (Fig. 6e, f)^43^. We also observed that Pbp1 15Q, 30Q, and 40Q exhibit decreased, but not abolished, interactions with ncRNAs from the IGS (Fig. 6g). Thus, SCA2-modeling Pbp1 proteins exhibit lower interactions with IGS ncRNAs and compromise rDNA repeat stability without altering Pbp1-dependent R-loop suppression or Sir2-dependent chromatin silencing.

In wild-type cells, RNA Pol II transcribes ncRNAs from an IGS promoter called E-pro (Fig. 6a)^27^. Hyper-induction of IGS ncRNA expression upon substitution of E-pro with a galactose-inducible promoter displaces cohesin complexes from the IGS, leading to increased rDNA repeat instability and a shortened replicative lifespan^27,33^. On the other hand, the complete shutoff of ncRNA transcription leads to a hyper-accumulation of cohesin across the entire IGS region and can extend replicative lifespan in some settings^33^. Considering the central role of IGS ncRNA expression in rDNA repeat and lifespan control, we asked if Pbp1 polyQ expansions alter ncRNA levels across the IGS. We found that Pbp1 15Q, 30Q, and 40Q cells, but not Pbp1 5Q or *pbp1Δ* cells, exhibit significant reductions in the levels of longer IGS1 ncRNAs (Fig. 6a and h). In addition, expression of a SCA2-linked ATXN2 polyQ in human cells similarly decreased the levels of IGS ncRNAs (Fig. 6i). Thus, SCA2-modeling polyQ forms can lower the levels of IGS ncRNAs.

### Protein aggregation disrupts rDNA/lifespan-regulatory ncRNAs

**Fig. 7:**
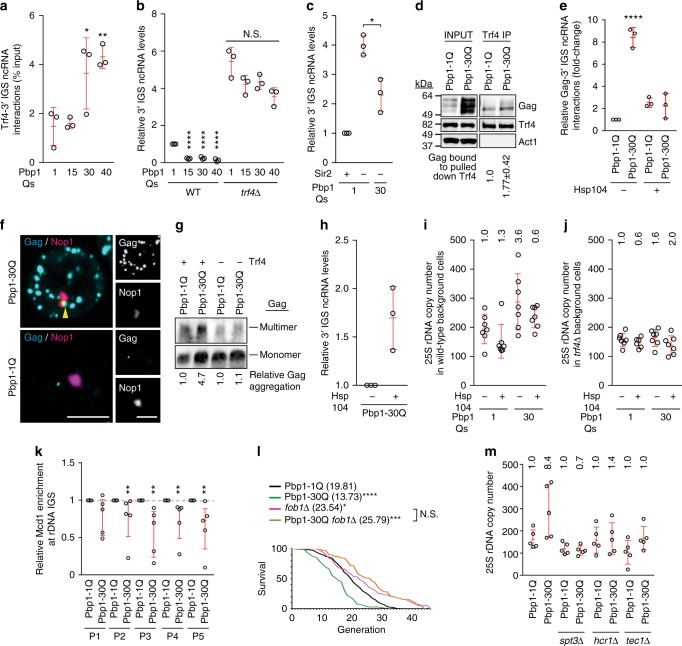
Protein aggregation compromises lifespan-regulatory ncRNA at rDNA repeats. **a** Interactions between Trf4 and IGS ncRNAs in Pbp1-polyQ cells. Presented are levels of IGS ncRNAs co-immunoprecipitating with Trf4 relative to input, as detected by RT-qPCR and normalized to mock IP signal from *trf4∆* cells (One-way ANOVA and Dunnett’s post hoc). **b** Effects of Pbp1-polyQ expansion on IGS ncRNAs in the presence/absence of Trf4, as detected by RT-qPCR (One-way ANOVA and Dunnett’s post hoc). **c** Effects of Pbp1-polyQ expansion on IGS ncRNAs in the presence/absence of Sir2, as detected by RT-qPCR (Student’s *t*-test). **d** CoIP examining the interaction between Trf4 and Gag (*n* = 2). **e** Interactions between Gag and IGS ncRNAs in Pbp1-polyQ cells. Presented are levels of IGS ncRNAs co-immunoprecipitating with Gag relative to input, as detected by RT-qPCR (One-way ANOVA and Dunnett’s post hoc). **f** Representative images showing the co-localization of Gag aggregates with nucleolar Nop1. Scale bar, 5 µm. **g** SDD-AGE of Gag in cells with/without Trf4. Multimer quantification relative to Pbp1-1Q cells is below the blot. **h** Effects of Hsp104 over-expression on IGS ncRNAs in Pbp1-30Q cells as detected by RT-qPCR. **i** Effects of Hsp104 over-expression on rDNA copy number in Pbp1-polyQ cells, as detected by qPCR (Mean ± SD; *n* = 7). **j** Effects of Hsp104 over-expression on rDNA copy number in Pbp1-polyQ cells without Trf4, as detected by qPCR (Mean ± SD; *n* = 7). **k** ChIP examining enrichment of the cohesin subunit Mcd1 at rDNA IGS (bars mark quartiles; *n* = 3; Mann–Whitney *U*-test). **l** Effect of blocking rDNA recombination on replicative lifespan in Pbp1-polyQ cells. Values are plotted as a survival curve, and mean lifespans are indicated in parentheses (Mann–Whitney *U*-test). **m** Effects of *spt3*∆, *hcr1*∆ and *tec1*∆ in rDNA copy number qPCR from Pbp1-30Q cells (Mean ± SD; *n* = 5). **a**–**c**, **e**, **h** Mean ± SD, *n* = 3. **a**–**c**, **e**, **k**–**l** Statistics are relative to Pbp1-1Q. **i**–**j**, **m** Variance is presented relative to Pbp1-1Q. **a**–**m** *p < 0.05; **p < 0.01; ***p < 0.001; ****p < 0.0001

In yeast, longer IGS ncRNAs are targeted for degradation by the Trf4 polyadenylase^30,32^. Thus, we asked whether SCA2-linked polyQ forms of Pbp1 hyperactivate Trf4-dependent turnover of longer IGS ncRNAs. Consistent with this model, RIP revealed a trend of increased interaction between Trf4 and longer IGS ncRNAs in Pbp1 15Q, 30Q, and 40Q cells (Fig. 7a). In addition, *trf4Δ* increased rDNA IGS ncRNA levels, as expected (Fig. 7b). Moreover, in the absence of Trf4, Pbp1 15Q, 30Q, and 40Q lost the ability to decrease the levels of longer IGS1 ncRNAs (Fig. 7b). This does not simply reflect the elevated levels of IGS ncRNAs in *trf4Δ* cells, since polyQ-expanded Pbp1 can decrease the elevated levels of these ncRNAs in *sir2Δ* cells (Fig. 7c). Thus, SCA2-linked Pbp1 polyQs hyperactivate Trf4-dependent turnover of longer IGS ncRNAs.

Next, we used Pbp1 30Q as a model to address whether Gag protein aggregation may be hyperactivating Trf4-dependent IGS ncRNA turnover and destabilizing rDNA repeats. First, we observed that 30Q increases the level of Gag co-immunoprecipitating with Trf4 (Fig. 7d). Then we found that 30Q cells display increased interactions between Gag and IGS ncRNA (Fig. 7e), suggesting that Gag aggregates could be involved in degrading the IGS ncRNAs and thus may localize to the nucleolus. Importantly, this increased interaction between Gag and IGS ncRNAs in 30Q cells is abolished upon Hsp104 over-expression (Fig. 7e). In addition, 30Q results in the accumulation of Gag-GFP aggregates throughout the cell and within the rDNA-harboring nucleolus (Fig. 7f; Supplementary Fig. 4). Moreover, Gag protein aggregation is dependent on Trf4 (Fig. 7g). In fact, over-expressing Hsp104 in 30Q cells increased the levels of IGS ncRNAs and rDNA copy number homogeneity (Fig. 7h, i). Interestingly, in the absence of Trf4, the ability of 30Q to increase and of Hsp104 over-expression to decrease rDNA copy number variance is greatly decreased (Fig. 7j). This is consistent with the finding that Gag aggregates are Trf4-dependent (Fig. 7g). This also suggests that the ability of Hsp104 over-expression to rescue rDNA copy number homogeneity is not due to unforeseen effects. However, we cannot rule out the possibility that the disaggregation of Gag and potentially unidentified factors may collectively restore rDNA stability in Pbp1 30Q cells. Together, these findings suggest that nucleolar Gag aggregation hyperactivates Trf4-dependent IGS ncRNA turnover, triggering rDNA instability.

Cohesin complexes typically repress rDNA instability by establishing various chromosomal structures^57^. Interestingly, cohesin enrichment at the rDNA IGS can be influenced by changes in IGS ncRNA levels. Thus, we asked whether the decreased levels of longer IGS ncRNAs in Pbp1 30Q cells are matched by changes in cohesin-IGS interactions. Therefore, we conducted ChIP experiments examining the enrichment of the cohesin subunit Mcd-1 across the IGS. 30Q cells exhibited decreased cohesin levels across the IGS (Fig. 7k). This indicates that the cohesin complex, whose IGS localization and rDNA-stabilizing function are known to be influenced by IGS ncRNA, is not efficiently loaded to the IGS in Pbp1 30Q cells.

Next, we asked whether it is the increased rDNA instability in Pbp1 30Q cells that underlies their shorter lifespan. Indeed, 30Q failed to shorten lifespan in the absence of Fob1, which is required for recombination within the rDNA repeats (Fig. 7l and Supplementary Table 2)^26,43–45,58^. Thus, these and previous lifespan experiments (Figs. 5j, 7l) suggest that lifespan shortening in 30Q cells requires rDNA instability and Ty1 expression, respectively. Thus, we deleted three key Ty1-promoting factors (Spt3, Hcr1, and Tec1) from wild type and 30Q cells before examining rDNA stability. Strikingly, deletion of Spt3, Hcr1, or Tec1 was sufficient to restore rDNA repeat stability in 30Q cells (Fig. 7m). Therefore, SCA2-modeling Pbp1 polyQ proteins and Trf4 interact more with each other and with Gag, inducing its self-aggregation in the rDNA-harboring nucleolus. This targets longer IGS ncRNAs for Trf4-dependent hyper-turnover resulting in rDNA instability and premature cellular aging.

## Discussion

Our results uncover a role for Pbp1 in Ty1 retrotransposition. We also reveal that Pbp1/ATXN2 loss and polyQ expansion disrupt transposons, rDNA repeat stability and lifespan via distinct mechanisms. In addition, we connect the major aging mechanisms of rDNA instability and protein aggregation.

**Fig. 8:**
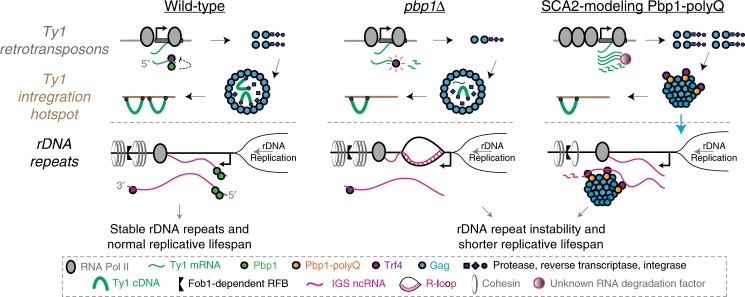
Proposed model. Pbp1 loss represses Ty1 retrotransposition by promoting Trf4-dependent degradation of Ty1 mRNA and increases rDNA instability via the accumulation of intergenic R-loops. SCA2-modeling Pbp1-polyQ proteins induce Gag expression and mediate Trf4-dependent *trans*-aggregation of Gag in the nucleolus, where Trf4 then induces the hyper-degradation of longer IGS ncRNAs that typically promote cohesin-IGS interactions. Consequently, cells exhibit rDNA instability and premature aging. See text for more details

Specifically, Pbp1 promotes retrotransposition by inhibiting Ty1 mRNA turnover, and ensures rDNA stability by restraining IGS R-loops (Fig. 8, left). Upon Pbp1 knockout, Ty1 retrotransposition is decreased and rDNA instability is increased (Fig. 8, middle). In SCA2-modeling cells, Pbp1 polyQ proteins and Trf4 interact with and promote the aggregation of Gag (Fig. 8, right). This dominantly inhibits retrotransposition despite decreasing Trf4-dependent turnover of Ty1 mRNA. In this context, nucleolar Gag aggregates induce Trf4-dependent hyper-turnover of longer IGS ncRNAs, resulting in rDNA repeat instability and premature aging.

We found that *pbp1Δ* and Pbp1 polyQ expansions respectively decrease and increase Ty1 mRNA levels. Similarly, human ATXN2 knockdown and polyQ expansions differentially impact abundant human retroelements, suggesting that our yeast genetic models can predict disease-linked molecular features in human cells. Directly contrasting ATXN2 loss and polyQ expansion is critical as ATXN2 repression is protective in certain mouse models of neurodegeneration while polyQ expansions appear to be always deleterious^59,60^. Importantly, mouse Atxn2 polyQ models have successfully contributed to our understanding of Ataxin-2 polyQ-related pathology despite the fact that mouse Atxn2, similar to yeast Pbp1, does not harbor a natural polyQ tract as in humans. Taken together, by bringing the power of yeast genetics to the study of ATXN2 polyQs, we hope to accelerate the identification of pathological processes and therapeutic avenues.

We favor the view that the actual number of Qs added, rather than the absolute length of the polyQ tract, to the wild-type Pbp1/ATXN2 protein guides their relevance to human disease. This is because these wild-type proteins already exhibit similar functions in different organisms despite having different baseline polyQ tract lengths. In addition, Pbp1 15Q, which has a polyQ tract that is shorter than that of wild-type human ATXN2, elicits selective loss-of-function phenotypes that are similar to those caused by Pbp1 30Q and 40Q. In addition, due to the overlap between ALS and SCA2 at moderate polyQ expansions, we cannot ascribe ALS-specific phenotypes in our study.

The destabilization of rDNA repeats is a major driver of replicative aging in yeast, and rDNA instability has been observed in physiological and pathological settings relevant to human health^5,33,34,43–45,58,61,62^. The deleterious aggregation of proteins has also emerged as a driver of cellular aging^46–48^. However, protein aggregation and rDNA instability models of aging are thought to be independent of each other. In contrast, our data connect Pbp1 polyQ expansion to Gag aggregation-dependent rDNA repeat instability and cellular lifespan shortening. First, polyQ expansion results in Gag aggregation, rDNA instability and lifespan shortening. Second, in the presence of polyQ expansion, Gag aggregation increases rDNA instability. This is supported by the repression of Gag aggregation and restoration of rDNA stability in polyQ-containing cells upon Trf4 knockout or Hsp104 over-expression. Third, in the presence of polyQ expansion, the repression of Gag or rDNA instability restores normal lifespan. This is supported by the fact that the lifespan of cells with polyQ expansion is fully rescued upon repressing Gag (via *tec1Δ*) or dominantly restoring rDNA stability (via *fob1Δ*). Fourth, repressing Gag via knockout of Spt3 or Hcr1 mimics the knockout of Tec1 and restores rDNA stability in cells with polyQ expansion. These findings connect the rDNA instability and protein aggregation models of aging.

Of note, we found that Hsp104 over-expression in wild-type cells does not decrease the variance in rDNA copy numbers but instead decreases the mean rDNA copy number by ~23% (Fig. 7i). This tentatively suggests that a baseline level of protein aggregation may allow for the establishment of a higher rDNA copy number in wild-type cells. Thus, protein aggregation may impact rDNA repeats in both pathological and non-pathological scenarios. Future studies should examine if wild-type Pbp1, or especially the polyQ-containing wild-type ATXN2, may aggregate with natural or physiological aging as part of an aging-dependent mechanism of retrotransposon inhibition.

The hyper-induction of IGS ncRNA displaces cohesin across the entire IGS, resulting in rDNA instability and lifespan shortening^27,33^. In contrast, the complete shutoff of IGS ncRNA results in the hyper-recruitment of cohesin across the IGS^27,33^. This can promote lifespan in certain settings. These previous studies differed from ours in that they examined the effect of totally shutting off IGS ncRNA expression, and this via changes to transcription rather than ncRNA turnover. Unlike these scenarios, SCA2-modeling Pbp1 polyQs induce intermediate phenotypes in which only some ncRNAs are decreased. These changes are accompanied by reductions in both cohesin recruitment and chromosome stability at rDNA repeats.

We uncover roles for a conserved protein impacting health in transposon control, create powerful yeast genetic models of human neurodegenerative diseases, and connect two major mechanisms of aging. Future work employing the herein created yeast genetic models of disease should provide unique insights into physiological and pathological mechanisms.

## Methods

### Yeast strain creation

All yeast transformations were carried out using standard lithium acetate-based protocols. Early log phase cells were resuspended in LiOAC mix (100 mM lithium acetate pH 7.3, 10 mM Tris-HCl, pH 8.0, 1 mM EDTA). A volume of 100 μL of cells were incubated with purified concentrated transforming DNA, 30 μL of salmon sperm DNA (10 µg/µL) and 700 μL PEG Mix (21 g PEG3350, 100 mM LiOAc, pH 7.3, 10 mM Tris-HCl, pH 8.0, 1 mM EDTA) at 30 °C for 45 min. Cells were subsequently treated with 100 μL of DMSO and incubated at 42 °C for 15 min. Cells were pelleted, resuspended in SOS mix (1 M sorbitol, 1/3 v/v YEP media, 6.5 mM CaCl_2_) and plated. For antibiotic resistance selection, cells were plated onto YEPD plates and transferred to antibiotic selection plates after 24 h. For amino acid selection, cells were plated directly onto dropout medium. Deletions were confirmed by colony PCR. All yeast strains used in this study are presented in Supplementary Data 1.

### Yeast cell culture

Yeast strains were grown in YEPD (20 g/L peptone; 10 g/L yeast extract; 2% glucose) or synthetic complete medium (2 g/L amino acid dropout powder; 6.67 g/L yeast nitrogen base; 2% glucose) to maintain plasmids. Strains were grown at 30 °C or 22 °C to induce Ty1 retrotransposition.

### Human cell culture

HEK293T cells were grown in Dulbecco’s Modified Eagle’s Medium (DMEM) with 10% fetal bovine serum (FBS) and 1% penicillin/streptomycin, and cultured in a 37 °C incubator with 5% CO_2_ in a humidified atmosphere.

### PolyQ plasmid creation

The vector pRS303 was used as a backbone for cloning. First, a TAP tag was cloned into the vector using the restriction enzymes PdiI and XhoI (Thermo Fisher Scientific). Next, a full length Pbp1 including the native promoter (460 bp upstream of Pbp1 start site) PCR product was generated from W303 genomic DNA using Phusion (NEB) and cloned into the TAP-tagged vector using PdiI and PauI (Thermo Fisher Scientific). To generate the polyglutamine plasmids, Pbp1 polyglutamine sequence plasmids (460 bp upstream to ClaI cut site within Pbp1 downstream of polyglutamine site) were ordered from Thermo Fisher Scientific. These plasmids were digested with ClaI and PauI (Thermo Fisher Scientific) and the Pbp1 polyglutamine sequence was gel purified and ligated into the full length Pbp1+ promoter plasmid. All plasmids had sequence fidelity confirmed by Sanger sequencing.

### Co-immunoprecipitation

Yeast strains were grown at 30 °C overnight, and then diluted 1:4 and grown to log phase at 30 °C (~3 h). An equivalent number of cells per strain (200 × 10^7^) were washed (50 mM Tris-HCl, pH 7.5, 150 mM NaCl), resuspended in lysis buffer (50 mM HEPES KOH, pH 7.5, 150 mM NaCl, 10% glycerol, 0.5% NP-40, 1 mM EDTA, 1 mM PMSF, cOmplete protease inhibitor) and disrupted with glass beads. Lysates were cleared and an aliquot was taken to serve as the input sample (diluted in lysis buffer and loading dye, then sheared with a 25G needle). Another aliquot of each lysate was used as the IP sample, and was incubated with IgG sepharose 6 fast-flow beads at 4 °C for 2 h (or 2 μg antibody for 2 h at 4 °C followed by incubation with Protein-A sepharose beads for 1 h at 4 °C for CoIPs of non-TAP-tagged proteins). Beads were then washed with lysis buffer to remove unbound protein, and then eluted by boiling in loading dye. Input and IP protein samples were analyzed following standard western blot protocols outlined below.

### Retromobility assay

Yeast strains were grown at 30 °C to saturation (overnight), then diluted 1:1000 and grown to saturation at 22 °C (3–5 overnights). Aliquots of saturated culture (100 μL to 1 mL) were plated onto SC-His (or SC-His-Ura for polyQ strains) plates, on which cells can only grow if they have undergone retrotransposition (His + cells). Aliquots of 1:100,000 and 1:1,000,000 dilutions were plated onto SC control (or SC-Ura for polyQ strains) plates to titer viable cells. Ty1 retromobility rate was calculated as the number of His^+^ cells divided by the total number of viable cells plated. Where possible, two clones of each strain were assessed. This assay monitors the ability of a single Ty1 element to move once. The *HIS* was swapped for *URA* in the pRS303 polyQ plasmids in order to ensure compatibility with this assay.

### DNA isolation

Cells were grown to saturation, centrifuged and washed with sterilized water. A volume of 200 μL of genomic lysis solution (2% Triton X-100, 1% SDS, 100 mM NaCl, 10 mM Tris-HCl, pH 8, 1 mM EDTA), 200 μL of phenol:chloroform:isoamyl alcohol (25:24:1), and 300 μL of glass beads were added to cells followed by bead-beating for 8 min at RT. The samples were centrifuged (16,000×*g*, 4 °C, 5 min) and DNA was precipitated by adding 1/10 volume of 3 M NaOAc, pH 5.2 and 2 volumes of cold 100% EtOH and centrifuged (16,000×*g*, 4 °C, 15 min). The resultant DNA pellet was washed with 2 volumes of cold 70% EtOH and let dry. The dried DNA pellet was resuspended in 200 μL TE (10 mM Tris-HCl, pH 8, 1 mM EDTA) and treated with 3 μL RNase (10 mg/ml) at 37 °C for 10 min.

### Ty1 integration PCR assay

Yeast strains were grown to saturation at 30 °C overnight, diluted 1:1000, and grown to saturation at 22 °C (3–5 overnights). DNA was phenol:chloroform extracted, washed with ethanol and eluted in TE. Concentrations were normalized to 1 μg/μL, and analyzed by standard PCR using primers within *TYB* (F) and *tRNA*^*GLY*^ (R) or *tRNA*^*TYR*^ (R) and F/R primers specific to *TEL1* as a PCR control. PCR reactions were performed for 25 cycles at 95 °C for 1 min, 60 °C for 30 s, 72 °C for 3 min (15 cycles for *TEL1* controls). Integration gel images presented in the main figures are cropped for clarity, full un-cropped gel images are presented in Supplementary Fig. 5, 8 and 9. This assay has the potential to monitor numerous Ty1 elements that may retrotranspose multiple times.

### ATXN2 plasmid transfections and knockdown experiments in human cells

For ATXN2 polyQ over-expression experiments, 4.5 × 10^5^ cells seeded in 6-well plates were transfected with 2 μg of plasmid using LipoD293 transfection reagent according to manufacturer’s instructions. For ATXN2 knockdown experiments, 3.5 × 10^5^ cells seeded in 6-well plates were transfected with a final in-media concentration of 10 nM siControl or siATXN2 using lipofectamine RNAiMax according to the manufacturer’s instructions. For both plasmid and siRNA knockdown experiments, RNA or protein was isolated from cells for subsequent interrogation by RT-qPCR or western blotting.

### RNA isolation

Yeast strains were grown to saturation at 30 °C overnight, diluted 1:4 and grown to log phase at 30 °C (Experiments determining Ty1 activity were conducted at 22 °C). Cells were pelleted and resuspended in buffer AE (50 mM NaOAc, pH 5 and 10 mM EDTA in 0.1% DEPC) and 1/10 volume of 10% SDS, then one volume of acid phenol (pH 4.5) was added. Samples were heated at 65 °C for 4 min, followed by 2 min in a dry ice-ethanol bath. RNA was then phenol:choloroform extracted, washed with ethanol and eluted in DEPC-treated H_2_O. RNA concentration was analyzed by NanoDrop and RNA was stored at −80 °C. For human cells, RNA was isolated from using the RNeasy Mini Kit (Qiagen) according to the manufacturer’s protocol.

### Reverse transcriptase qPCR

A volume of 1 μg of total RNA was DNase I-treated, heat-inactivated and then reverse-transcribed following manufacturer’s protocols. Briefly, a 20 µL RT reaction was carried out using 10 mM dNTPs, 50 µM random nonamers (Sigma), 1 μg DNase I (Invitrogen) treated RNA, 5X FirstStrand Buffer (Invitrogen), 100 mM DTT, 40 U/µL RNaseOUT (Invitrogen), and 200 U/µL MMLV reverse-transcriptase (Invitrogen) at 23 °C for 10 min, 37 °C for 60 min, and 70 °C for 15 min. Resulting cDNA was diluted 1:4 and qPCR was performed at 1 cycle of 95 °C for 5 min, 60 °C for 30 s, followed by 39 cycles of 95 °C for 5 s, 60 °C for 30 s. Results were analyzed using the following formula: ΔΔCt = 2^-(ΔCtMutant-ΔCtWT) where ΔCt = Ct (Gene)–Ct (control). Yeast experiments were normalized to *ACT1* and human experiments were normalized to GAPDH, unless otherwise stated. Our Ty1 RT-qPCR primers do not differentiate between full-length and truncated Ty1 RNA.

### Chromatin immunoprecipitation

Yeast strains were grown at 30 °C overnight, and then diluted 1:4 and grown to log phase at 30 °C (Experiments determining Ty1 activity were conducted at 22 °C). An equivalent number of cells per strain (200 × 10^7^) were cross-linked for 15–30 min (1% formaldehyde, gentle shaking). Cells were washed (20 mM Tris-HCl, pH 7.6, and 150 mM NaCl), resuspended in lysis buffer (50 mM HEPES KOH, pH 7.5, 500 mM NaCl, 1 mM EDTA, 1% Triton X-100, 0.1% sodium deoxycholate, 0.1% SDS, 1 mM PMSF, cOmplete protease inhibitor) and disrupted with glass beads. Resulting lysates were sonicated for 20 s at 40% amplitude (three times with 2 min intervals on ice). Lysates were cleared and an aliquot was taken to serve as the input sample. Another aliquot of each lysate was incubated with 2 μg of antibody at 4 °C for 2 h, then Protein-A sepharose beads were added and incubation continued for another hour. The beads were then successively washed in lysis buffer, lithium chloride buffer (10 mM Tris-HCl, pH 8, 250 mM LiCl, 0.5% NP-40, 0.5% sodium deoxycholate, 1 mM EDTA) and TE (10 mM Tris-HCl, pH 8, 1 mM EDTA) to remove unbound DNA, and bound DNA was eluted from the beads (IP sample) using elution buffer (50 mM Tris-HCl, pH 8, 10 mM EDTA, and 1% SDS). Input and IP samples were reverse-cross-linked at 65 °C overnight. Each sample was then incubated at 37 °C for 30 min with 0.2 μg/μL RNaseA, and for 2 h with 0.03 μg/μL glycogen and 0.2 μg/μL proteinase K to remove contaminating RNA and protein. DNA was then extracted by standard phenol:chloroform methods and analyzed by qPCR, parameters as described above. For qPCR analyses, IP signals were divided by Input signals and normalized to CUP1 controls, using the following calculations: [100*2^(Adjusted Input_Gene_–Ct (IP_Gene_))]/[100*2^(Adjusted Input_control_–Ct (IP_control_))], where Adjusted Input = Ct_Input_–log_2_1000. (normalized to *CUP1* unless otherwise stated).

### Protein isolation

Yeast strains were grown to saturation at 30 °C overnight, and then diluted 1:4 and grown to log phase at 30 °C (Experiments determining Ty1 activity were conducted at 22 °C). Protein was isolated using standard TCA-based protocols. Briefly, cell pellets (2.5 × 10^7^ cells) were washed with cold H_2_O, resuspended in H_2_O and incubated with Alkali/β-mercaptoethanol solution (1.85M NaOH, 1.065 M β-mercaptoethanol) on ice for 10 min, followed by the addition of 50% TCA solution and an additional 10 min incubation on ice. Lysates were pelleted and resuspended in loading buffer (1X standard loading buffer, 1.42M β-mercaptoethanol, 83.2 mM Tris-HCl, pH 8.8), boiled and re-pelleted. Supernatants were stored at −20 °C and boiled at 95 °C for 5 min prior to gel loading. For protein isolation from human cells, adherent HEK293T cells in 6-well plates were gently washed with ice cold 1X PBS, lysed using RIPA lysis buffer (10 mM Tris-Cl, pH 8.0, 1 mM EDTA, 1% Triton X-100, 0.1% sodium deoxycholate, 0.1% SDS, 140 mM NaCl, 1 mM PMSF) and kept on ice for 10 min. Lysates were then spun at 4 °C to remove debris, the supernatant was collected, and then sheared at least 5 times using a 26G needle. All protein and RNA samples were stored at −80 °C.

### Western blot

Proteins were separated on an 8% acrylamide gel and electrophoretically transferred to a nitrocellulose membrane. Ponceau staining was conducted to ensure even transfer. Membranes were washed in 1X TBST and blocked with 2.5% or 5% milk, then washed with 1X TBST and incubated at 4 °C with primary antibodies (1:500–1:5000) overnight. Membranes were washed in 1X TBST and incubated with secondary antibodies (1:2000) for one hour at RT. Membranes were washed in 1X TBST, incubated with ECL substrate and imaged via autoradiography or digital imaging using a VersaDoc imager. Act1 is presented as a loading control (unless otherwise stated). Blot images presented in the main figures are cropped for clarity, full un-cropped blot images are presented in Supplementary Fig. 6–9 and Supplementary Fig. 11–12. For Gag western blots, two antibodies were used to p22/18 on gels (Supplementary Fig. 2)^17^. Pbp1 loss and polyQs only altered p45/49.

### RNA immunoprecipitation

Lysates were prepared as in ChIP. Lysates were cleared and treated with 15 μg DNase I from bovine pancreas. An aliquot was taken to serve as the input sample. Another aliquot of each lysate was incubated with either IgG sepharose 6 fast-flow beads at 4 °C for 2 h (TAP RIP) or 2 μg of antibody at 4 °C for 2 h then added to Protein-A sepharose beads and incubated at 4 °C for an additional hour (Trf4 and Myc RIPs). The beads were then washed to remove unbound RNA, and then bound RNA was eluted from the beads (IP sample). Input and IP samples were reverse-cross-linked at 65 °C overnight. Each sample was then incubated with 0.2 μg/μL proteinase K to remove contaminating protein. RNA was then extracted by standard acid phenol methods, DNase I-treated and analyzed by RT-qPCR using the ΔΔ-Ct method as described above (normalized to *ACT1* unless otherwise stated) with 5′ (TYA) and 3′ (TYB2) primers for Ty1 mRNA and 3′ (P1 and P2) primers for IGS ncRNA. Our RIP experiments may detect direct and indirect binding.

### RNA stability assay

Yeast strains were grown to saturation at 30 °C overnight. Saturated cultures were diluted in 25 mL fresh medium and grown to log phase. Samples were treated with the RNA polymerase inhibitor, 1,10-phenanthroline, to a final concentration of 100 μg/mL. A volume of 5 mL aliquots were collected before treatment and at different time points post-treatment. RNA extraction and RT-qPCR were performed as described above.

### Semi-denaturing detergent agarose gel electrophoresis

Yeast strains were grown at 30 °C overnight, then diluted 1:4 and grown to log phase at 30 °C (~3 h). An equivalent number of cells per strain (200 × 10^7^) were washed (50 mM Tris-HCl, pH 7.5, 150 mM NaCl), resuspended in lysis buffer (50 mM HEPES KOH, pH 7.5, 150 mM NaCl, 10% glycerol, 0.5% NP-40, 1 mM EDTA, 1 mM PMSF, cOmplete protease inhibitor), and disrupted with glass beads. Lysates were cleared and 4X loading dye was added to a final 1X concentration. Samples were loaded on a 1.5% agarose gel with 0.1% SDS and electrophoresed at 4 °C in 1X TAE running buffer with 0.1% SDS at 30 V for ~18 h. Proteins were transferred to a nitrocellulose membrane by capillary transfer using 1X TBS. Membranes were then processed by standard western blotting techniques. The wild-type Gag-Pol fusion protein and virus-like particle are 199 kDa and 14 MDa, respectively. These are different from our observed moderate Gag aggregates, which are around 300 kDa.

### Replicative lifespan analysis

Yeast strains were grown on YEPD plates for 48 h at 30 °C. A small streak of cells was diluted in H_2_O and plated onto fresh YEPD plates. Using a dissecting microscope, single cells (*n* = 20) were arranged in a vertical line. Plates were incubated at 30 °C for 1.5 h to allow cells to bud. Upon budding, mother cells were removed and virgin daughter cells were left in place. Plates were incubated at 30 °C for 1.5 h to allow cells to bud. Upon budding, daughter cells were removed, discarded, and counted, while mother cells were left in place. Plate incubation and removal/counting of daughter cells were repeated until mother cells senesced. Experiments were repeated four times per strain to achieve *n* = 80. The replicative lifespan of each cell was represented by the number of times it budded, and an average was taken for all 80 cells.

### Copy number qPCR

For copy number determination, genomic DNA was serially diluted to 0.05 ng/μL. qPCR was performed using the Biorad CFX Connect Real-Time system. Primers within 25S rDNA were used to determine rDNA copy number and ACT1 was used as a single-copy reference gene.

### Contour-clamped homogenous electric field electrophoresis

Yeast strains were grown at 30 °C overnight, and 1 mL of saturated culture was washed and resuspended in TE (50 mM EDTA, 10 mM Tris-HCl, pH 7.5). Cells were lysed with 2 μL (20 μL/mL in 10 mM Na_2_HPO_4_, pH 7.5) zymolyase. A volume of 500 μL of low melting-point Seakem agarose (1% in 125 mM EDTA, pH 8, 42 °C) was subsequently added to cultures which were poured into plug molds and solidified at 4 °C. Plugs were incubated overnight sequentially in 10 mM Tris-HCl, pH 7.5, 500 mM EDTA (37 °C) and 1 mL 2 mg/mL proteinase K in 10 mM Tris-HCl, pH 7.5, 500 mM EDTA, 10 mg/mL N-lauroylsarcosine (50 °C). Plugs were washed three times with TE (4 °C, 1 h per wash) and stored in TE (4 °C). Plugs were cut to 5 × 3 × 1.5 mm and resolved on a 0.8% Seakem Gold agarose gel in 0.5X TBE using the CHEF-DR-II apparatus. CHEF running conditions were 68 h, 3.0 V/cm, 300–900 s, 10 °C. Gels were EtBr-stained and imaged, then capillary transferred to a nylon membrane using 20X SSC following standard protocols. Membranes were UV cross-linked for 5 min and primed in GE Rapid Hybridization buffer for >20 min. An IGS1 probe was created and α-32P-labelled using the GE Megaprime system. Primed membranes were then incubated with the radiolabeled probe overnight at 65 °C. Membranes were washed with 2X SSC 0.1% SDS, 1X SSC 0.1% SDS for 20 min at 65 °C sequentially followed by washes with 0.1X SSC 0.1% SDS until membrane radioactivity was below 1.8 Bq/cm². Nylon membranes were transferred to autoradiography films in a phosphorimager cassette with intensifying screens at −80 °C for 1–3 days and developed. Blot images presented in the main figures are cropped for clarity, full un-cropped blot images are presented in Supplementary Fig. 10.

### Live cell microscopy

Log phase cells were collected via centrifugation, washed once with dH_2_O and resuspended in dropout medium before mounting on a slide for image acquisition^63,64^. Asynchronous cells were subjected to live cell microscopy. Single plane images were taken with a Nikon C2+ Confocal Microscope using a Plan-Apochromat TIRF ×100 oil objective (numerical aperture 1.45) and processed with NIS-Elements AR (Nikon). Images were captured with excitation wavelengths of 405 nm and 488 nm with a 30–40 nm pinhole.

### Quantification and statistics

Statistical analyses were carried out using GraphPad Prism 7. Datasets were compared using two-tailed *t*-tests and one-way ANOVAs, or their non-parametric equivalents. Following ANOVAs, appropriate post hoc analyses were conducted depending on desired comparisons. Statistics are represented in the figures by asterisks, and statistical tests used are described in the figure legends. Results were considered significant if *p* < 0.05 (*p < 0.05, **p < 0.01, ***p < 0.001, ****p < 0.0001). “*n*” values represent the number of biological replicates, defined as the number of times an experiment was independently carried out. For retromobility assays, the five biological replicates consisted of two to three biological replicates from two independent transformants. For lifespan analyses, which were carried out in four biological replicates (each consisting of 20 technical replicates), “*n*” represents total number of cells.

### Key reagents

Key reagents such as antibodies and commercial kits are presented in Table 1.

**Table 1:** List of materials used in this study

Material	Company	CAT#
Antibodies		
Mouse monoclonal anti-Actin	Thermo Fisher Scientific (Invitrogen)	MA1-744
Rabbit anti-Gag	D. Garfinkel	N/A
Mouse monoclonal anti-Ty1-Tag	Sigma-Aldrich	SAB4800032
Rabbit peroxidase anti-peroxidase	Sigma-Aldrich	P1291
Mouse monoclonal anti-PABP1	EnCor Biotech	MCA-1G1
Rabbit anti-Trf4	D. Tollervey	N/A
Mouse monoclonal anti-RNA Pol II (Phosphorylated Serine 5)	AbCam	ab5408
Mouse monoclonal S9.6 (anti-RNA–DNA Hybrid)	KeraFast	ENH002
Mouse monoclonal anti-MYC	Covance research	MMS-150R-500
Mouse monoclonal anti-MYC HRP	Thermo Fisher Scientific (Invitrogen)	R951-25
Rabbit polyclonal anti-acetyl histone H3	Millipore Sigma	06-599
Rabbit polyclonal anti-histone H3	AbCam	Ab1791
Rabbit polyclonal Anti-ATXN2	Proteintech	21776-1-AP
Mouse monoclonal anti-ɑ-tubulin	Sigma	T6074

Chemicals		
1,10-phenanthroline	Sigma-Aldrich	131377-25G
DNase I (Bovine Pancreas)	Sigma-Aldrich	D7291
SensiFAST SYBR No-ROX Kit	BIOLINE	BIO-98050
Pierce ECL western blotting substrate	Thermo Fisher Scientific	32106
cOmplete protease inhibitor (Roche)	Sigma-Aldrich	11697498001
IgG sepharose 6 fast flow beads	Sigma-Aldrich	GE17-0969-01
Protein-A sepharose beads	GE Healthcare	17078001
Lipofectamine RNAiMAX	Thermo Fisher Scientific	13778150
LipoD293	Signagen	SL100668
SeaKem LE agarose	Lonza	50001

Commercial Assays		
Amersham megaprime DNA labeling system	GE Healthcare	RPN1606
M-MLV reverse transcriptase kit	Thermo Fisher Scientific (Invitrogen)	28025013
RNeasy mini kit	Qiagen	74104
KAPA2G Fast HotStart PCR Kit	KAPA Biosystems	KK5517

Oligonucleotides		
siRNA targeting ATXN2	Ambion	4390843
siRNA control	Ambion	4392420
TYA: ATCTATGATTCCGTATACAC and AGGATGAATCAGTAAATGTA	This study	N/A
TYB: AGAATACCGAGGAATCTATCATCGC and AGTCACCAATACCACCCAAACTG	This study	N/A
TYB-2: GACCGTGGTTCTGAGTATACT and TTCGGTAAACCACTACATTG	This study	N/A
Ty1His3: GGCCGTGCGTGGAGTAAAAA and AAGAAAATGCGGGATCATCTC	This study	N/A
Integration US *tRNA*^*GLY*^: GTGATGACAAAACCTCTTCCG and GGCAACGTTGGATTTTACCAC	^65^	N/A
Integration US *tRNA*^*TYR*^: GTGATGACAAAACCTCTTCCG and AGTCTTGCGCCTTAAACCAA	^65^ and this study	N/A
TEL1: CGGATTTCTGACGATATGGAC and ACCAACGTACTGAAGGTATCC	^65^	N/A
Cup1: TGAAGGTCATGAGTGCCAAT and TTCGTTTCATTTCCCAGAGCA	^34^	N/A
Act1: GCCTTCTACGTTTCCATCCA and GGCCAAATCGATTCTCAAAA	^34^	N/A
Pbp1: AAACCGATCAGCAAAACCCC and CCGGTAGATGATTGGCCTTG	This paper	N/A
IGS P1: CCGGGGCCTAGTTTAGAGAG and ACCCATCTTTGCAACGAAAA	^34^	N/A
IGS P2: AGGGCTTTCACAAAGCTTCC and TCCCCACTGTTCACTGTTCA	^34^	N/A
IGS P3: TGATGATGGCAAGTTCCAGA and CTTATTCCTTCCCGCTTTCC	^34^	N/A
IGS P4: GGAAAGCGGGAAGGAATAAG and CGATTCAGAAAAATTCGCACT	^34^	N/A
IGS P5: GTTGGTTTTGGTTTCGGTTG and TCGCCGAGAAAAACTTCAAT	^34^	N/A
25S rDNA: GGGAATGCAGCTCTAAGTGG and ATGGATTTATCCTGCCACC	This study	N/A
ATXN2: CTCCTCGGTGGTCGCGGCGACCTC and CTCTTTTTGCATAACTGGAGTCC	^66^	N/A
GAPDH: TGAAGGTCGGAGTCAACGGATTTGG and GGAGGCCATGTGGGCCATGAG	^66^	N/A
RPLP0: GGCGACCTGGAAGTCCAACT and CCATCAGCACCACAGCCTTC	^67^	N/A
LINE-1: TGCGGAGAAATAGGAACACTTTT and TGAGGAATCGCCACACTGACT	^68^	N/A
HERV-H: CACGTTTTATCCGTGGACCC and AGGCATCCCTGCAATGATTAA	^68^	N/A
Alu: TAGCCAGGTGTGGTGACTTG and AATGGTACGATCTFGGCTCA	^68^	N/A

Recombinant DNA		
p426 Empty GPD URA Vector (pKM55)	ATCC	ATCC87361
p426 GPD Hsp104 URA (pKM302)	^56^	Duennwald lab
p416 GAL Pbp1-1Q URA (pKM309)	This study	N/A
p416 GAL Pbp1-5Q URA (pKM310)	This study	N/A
p416 GAL Pbp1-15Q URA (pKM311)	This study	N/A
p416 GAL Pbp1-30Q URA (pKM312)	This study	N/A
p416 GAL Pbp1-40Q URA (pKM313)	This study	N/A
p416 Empty GAL URA Vector (pKM279)	This study	N/A
ATNX2-23Q-Halo (pKM286)	^55^	N/A
ATXN2-31Q-Halo (pKM288)	^55^	N/A
ATXN2-39Q-Halo (pKM291)	^55^	N/A
pRS303 Empty Vector HIS	ATCC	ATCC77138
pRS303 Pbp1-1Q-TAP HIS	This study	N/A
pRS303 Pbp1-5Q-TAP HIS	This study	Thermo Fisher Scientific
pRS303 Pbp1-15Q-TAP HIS	This study	Thermo Fisher Scientific
pRS303 Pbp1-30Q-TAP HIS	This study	Thermo Fisher Scientific
pRS303 Pbp1-40Q-TAP HIS	This study	Thermo Fisher Scientific
pLTR:Gag_1-401_-GFP LEU (pKM353)	^69^	Curcio lab
pRS316 Nop1-CFP URA (pKM354)	This study	N/A

## Electronic supplementary material

Supplementary file

Supplementary Data 1

Description of Additional Supplementary Files

## Data Availability

Sequence data have been deposited in NCBI’s GenBank and can be accessed via accessions MH986601, MH986602, MH986603, MH986604, and MH986605.
