# Supplementary material for: Conserved Pbp1/Ataxin-2 regulates retrotransposon activity and connects polyglutamine expansion-driven protein aggregation to lifespan-controlling rDNA repeats
Source: Commun Biol. 2018 Nov 5;1:187. doi: 10.1038/s42003-018-0187-3 (PMC6218562; doi:10.1038/s42003-018-0187-3)
Supplement: Supplementary file 1 — Supplementary file [file 42003_2018_187_MOESM1_ESM.pdf]

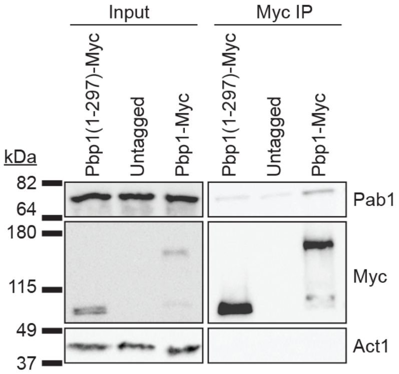

Supplementary Fig. 1, related to Fig. 1. Deletion of the Pab-1-binding domain of Pbp1 abolishes its interaction with Pab1. CoIP analysis examining the interaction between a Pbp1 truncation mutant lacking the C-terminal Pab-1-binding domain and Pab1. Act1 is presented as a loading control.

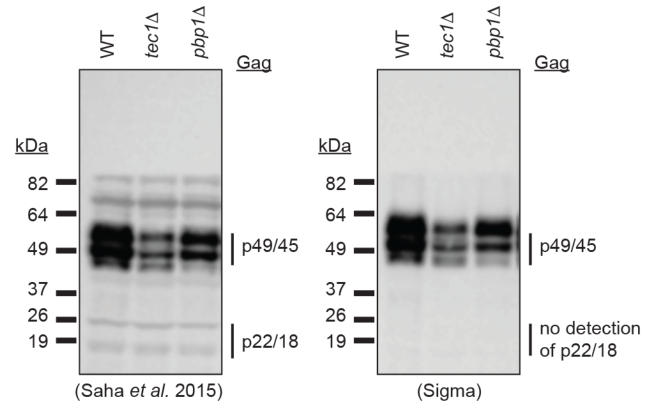

Supplementary Fig. 2, related to Fig. 2. Additional controls related to Gag western blotting. Western blots confirming the antibody (left) used in Fig. 2m can recognize the p49/45 and p22/18 forms of Gag.

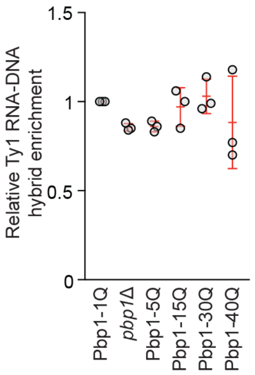

Supplementary Fig. 3, related to Fig. 6. Additional controls related to the impact of Pbp1 polyQ expansions on R-loops. Pbp1 polyQ expansions do not trigger R-loop accumulations at Ty1. Shown are the results of anti-RNA-DNA hybrid ChIP experiments (*n* = 3 biological replicates).

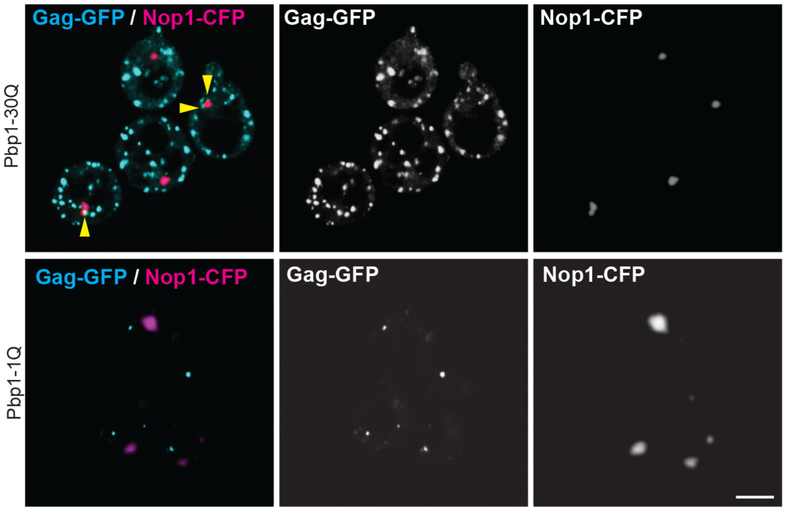

Supplementary Fig. 4, related to Fig. 7. Impact of Pbp1 polyQ expansions on Gag-GFP localization relative to nucleolar Nop1-CFP marker. Scale bar, 5 µm.

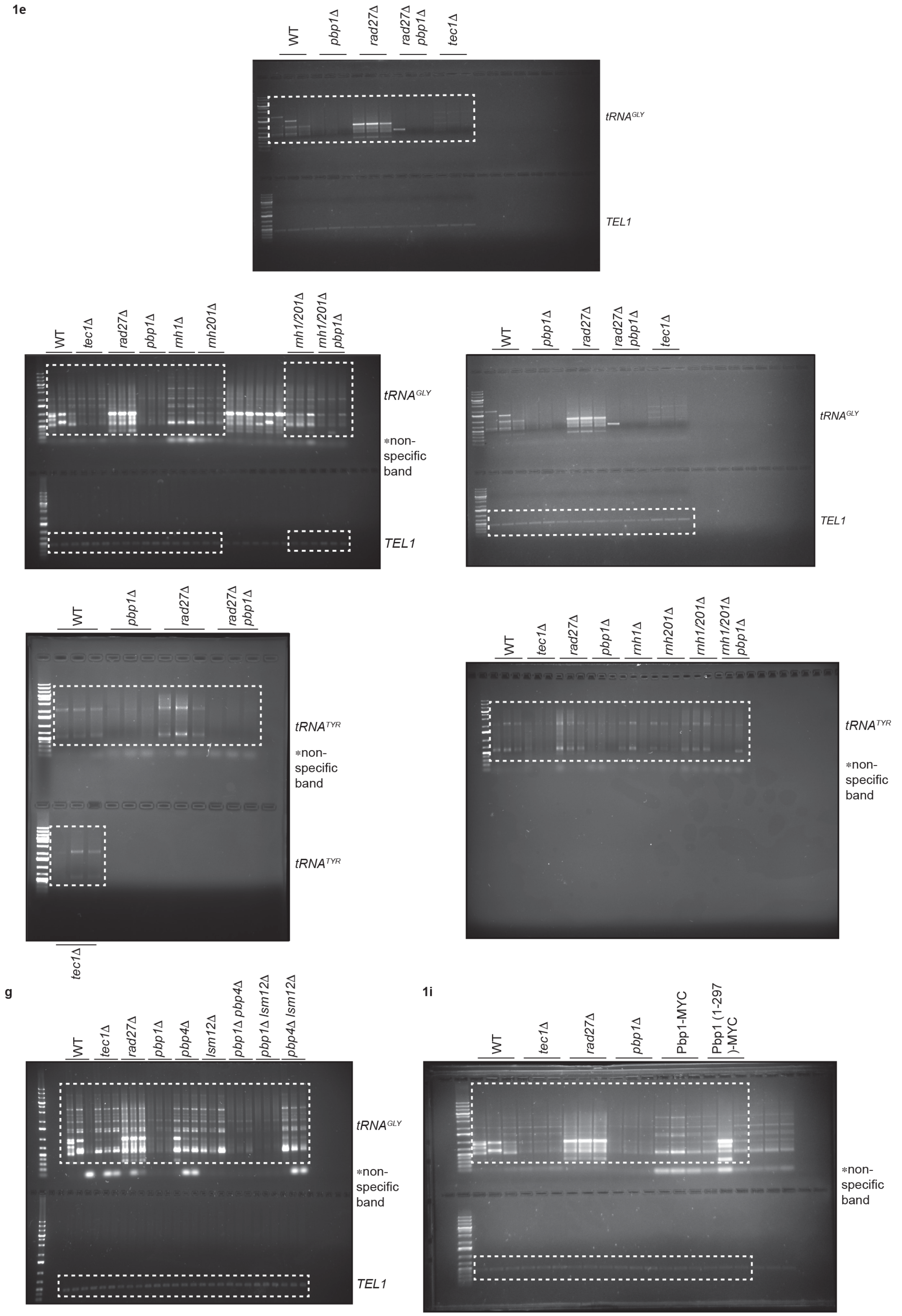

Supplementary Fig. 5, related to Fig. 1. Un-cropped gels and blots. Panels labeled as in Fig. 1.

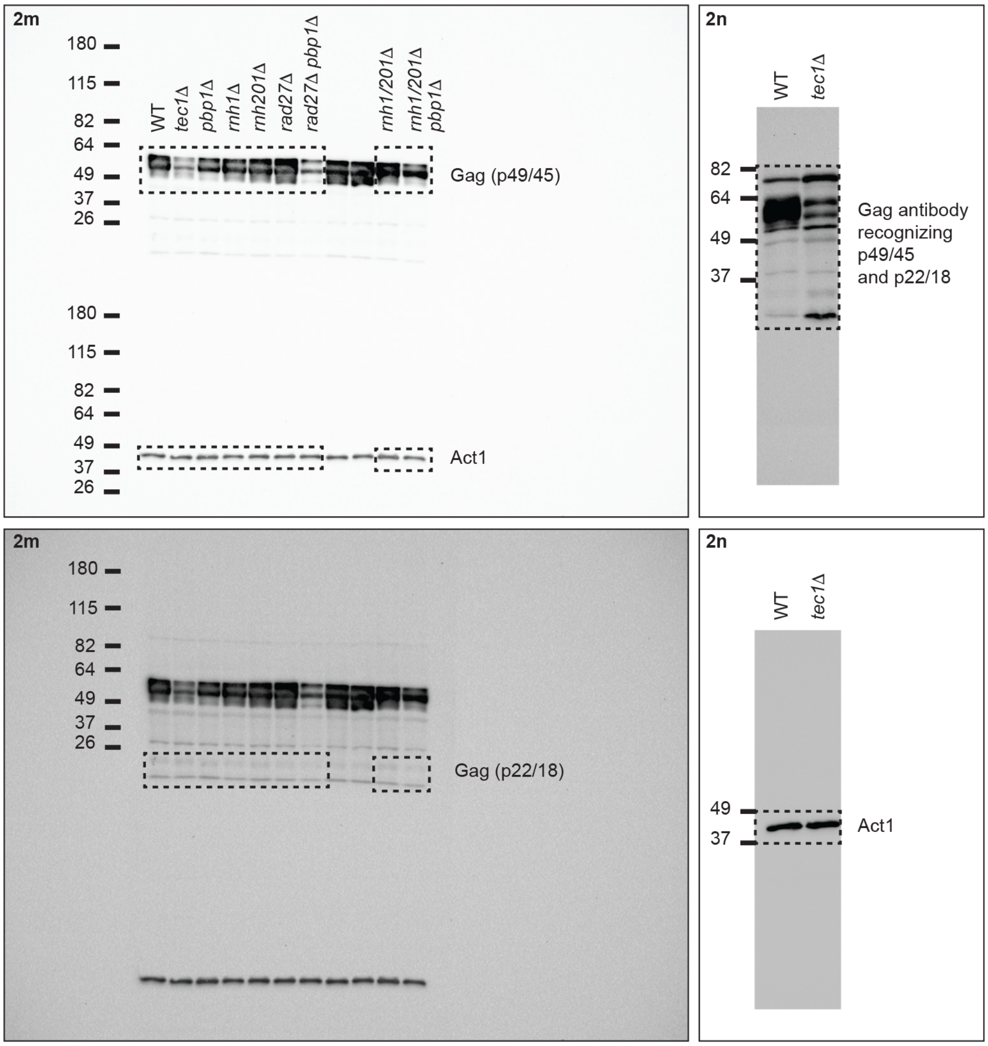

Supplementary Fig. 6, related to Fig. 2. Un-cropped gels and blots. Panels labeled as in Fig. 2.

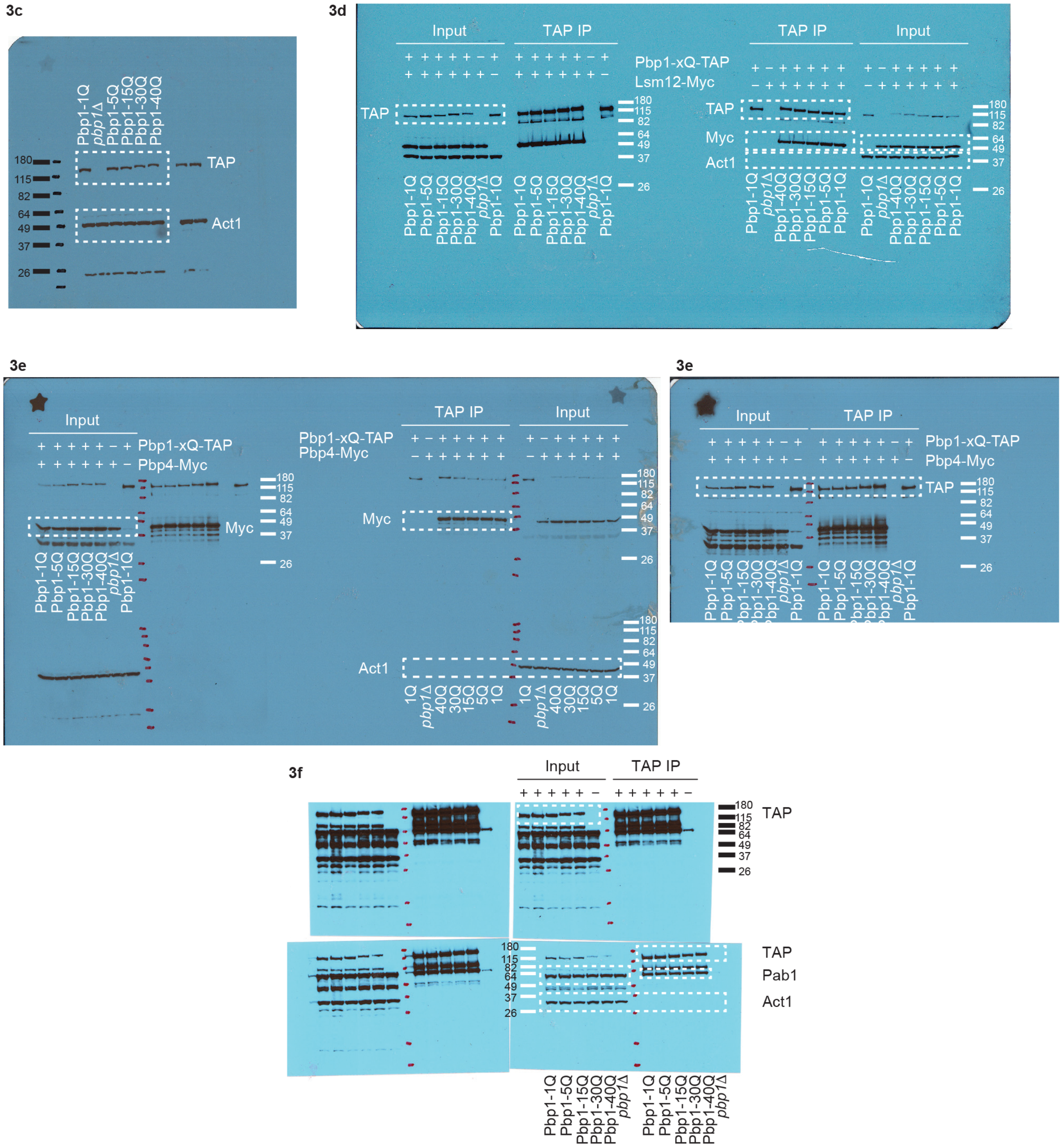

Supplementary Fig. 7, related to Fig. 3. Un-cropped gels and blots. Panels labeled as in Fig. 3.

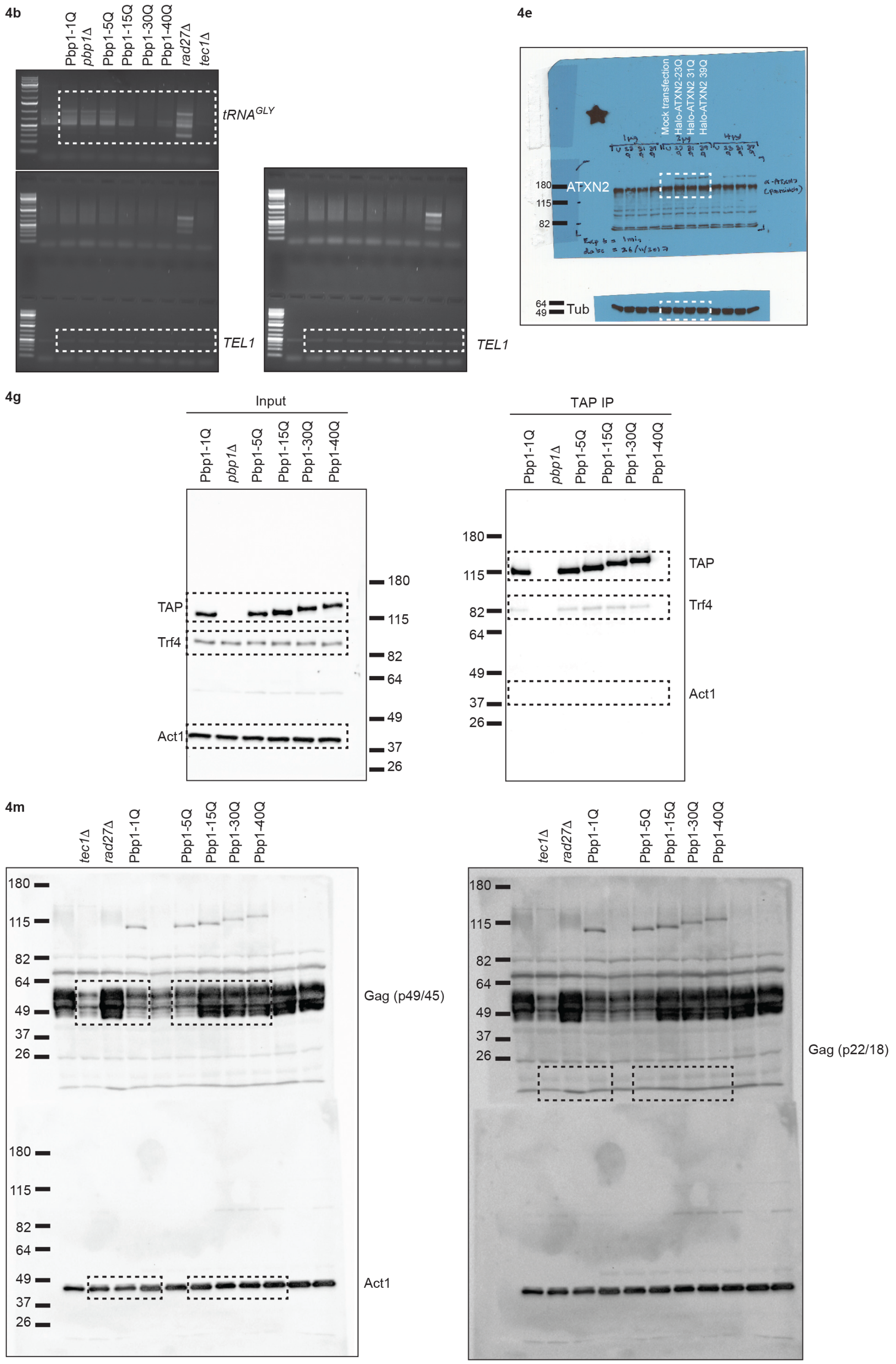

Supplementary Fig. 8, related to Fig. 4. Un-cropped gels and blots. Panels labeled as in Fig. 4.

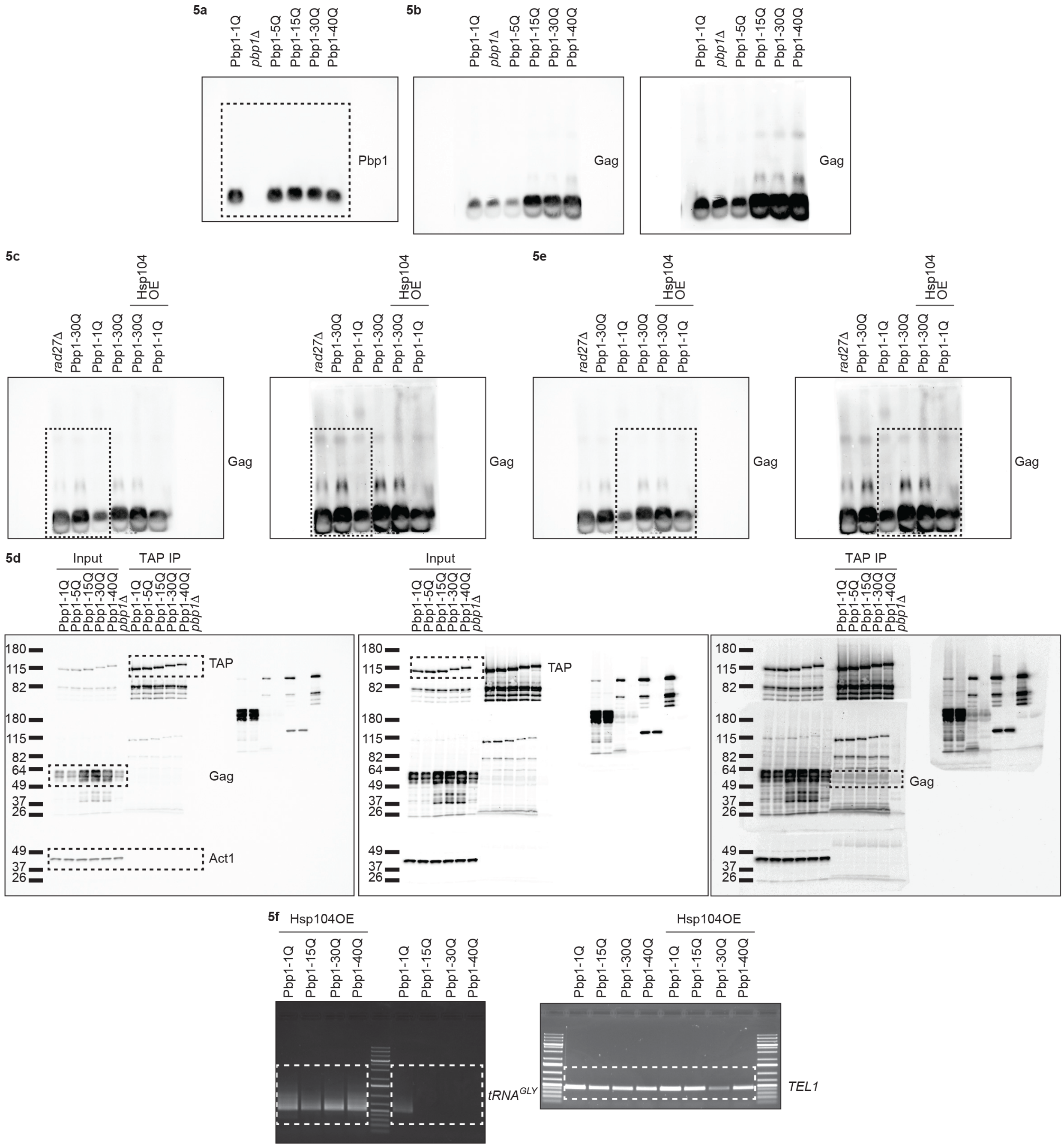

Supplementary Fig. 9, related to Fig. 5. Un-cropped gels and blots. Panels labeled as in Fig. 5.

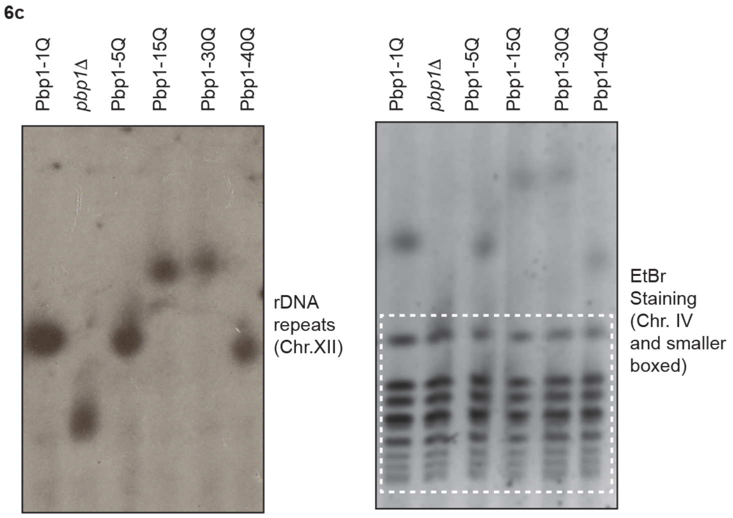

Supplementary Fig. 10, related to Fig. 6. Un-cropped gels and blots. Panels labeled as in Fig. 6.

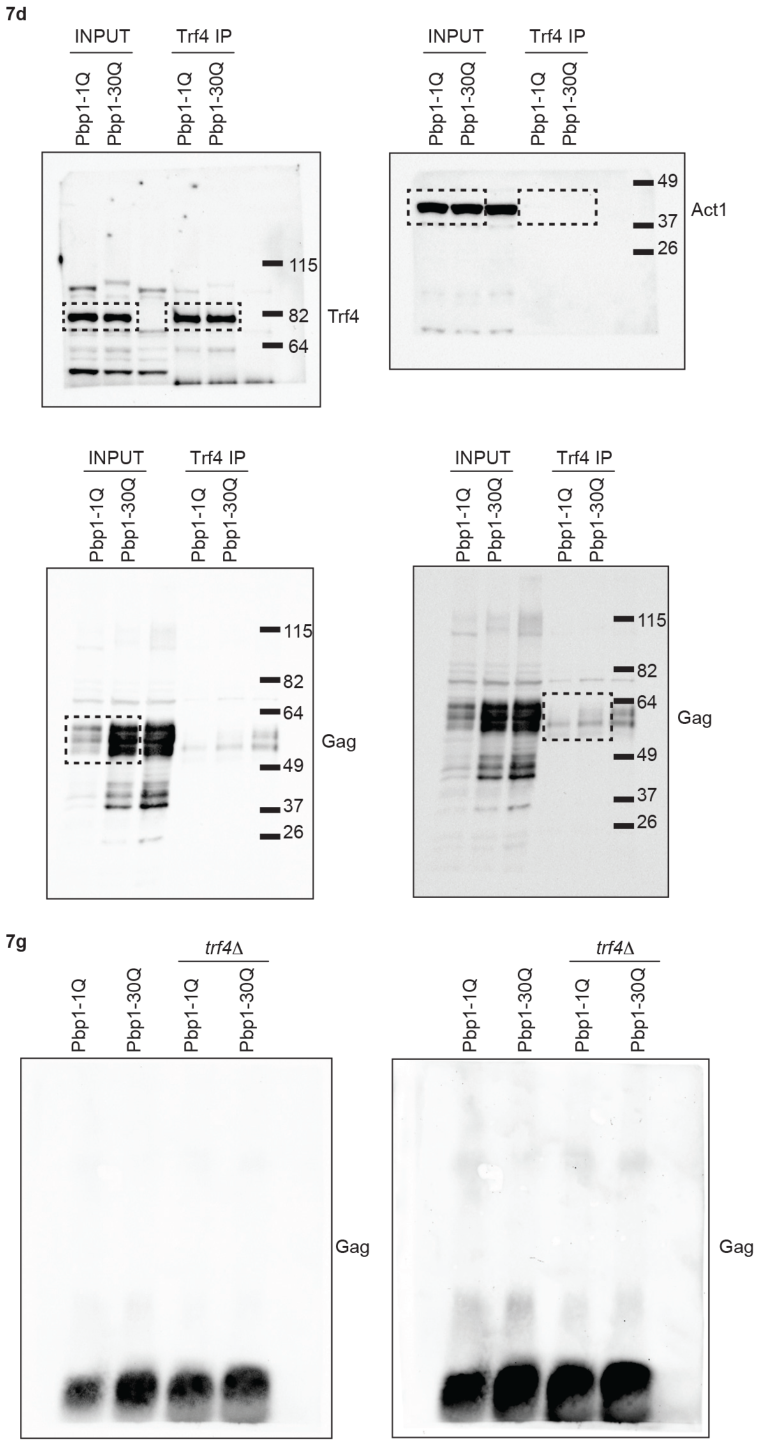

Supplementary Fig. 11, related to Fig. 7. Un-cropped gels and blots. Panels labeled as in Fig. 7.

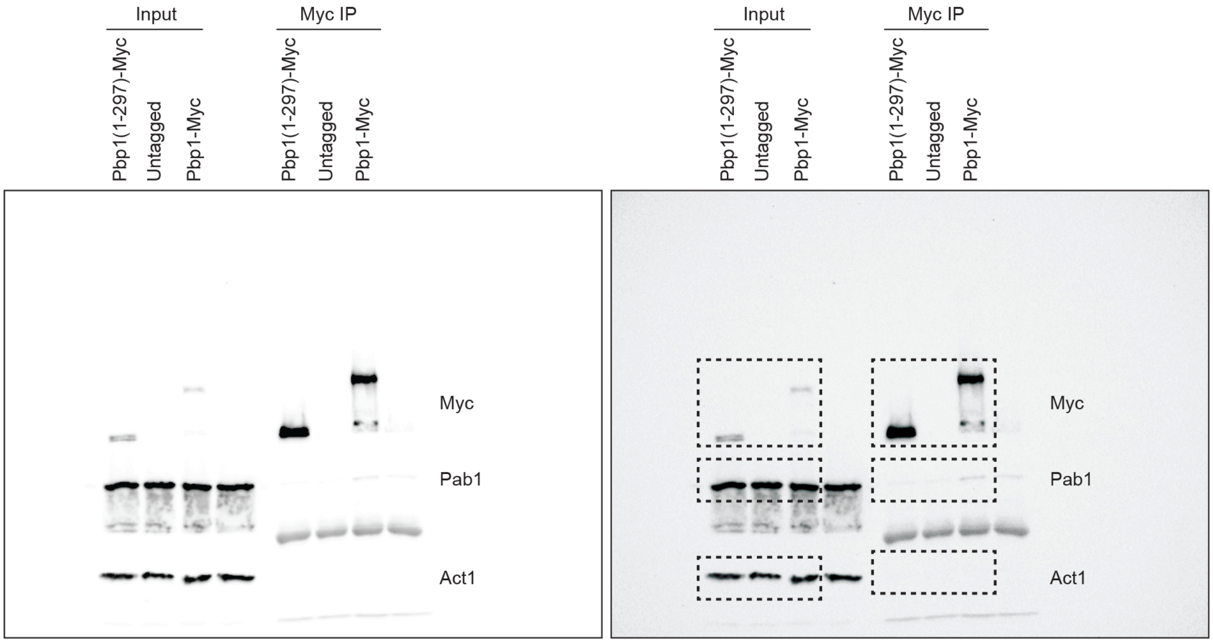

Supplementary Fig. 12, related to Supplementary Fig. 1. Un-cropped gels and blots. Panels labeled as in Fig. 11.

Supplementary Table 1, related to Fig. 5. Replicative lifespan measurements and *p* values for Mann-Whitney *U* test.

**Fig.**	**Strain A**	**Strain B**	***p* value**
5i	Pbp1-1Q (19.81, *n* = 200)	*pbp1Δ* (16.11, n = 80)	< 0.0001
	Pbp1-1Q (19.81, *n* = 200)	Pbp1-15Q (14.43, *n* = 79)	< 0.0001
	Pbp1-1Q (19.81, *n* = 200)	Pbp1-30Q (13.73, *n* = 98)	< 0.0001
	Pbp1-1Q (19.81, *n* = 200)	Pbp1-40Q (14.16, *n* = 80)	< 0.0001
	pbp1Δ (16.11, n = 80)	Pbp1-15Q (14.43, *n* = 79)	0.0741
	pbp1Δ (16.11, n = 80)	Pbp1-30Q (13.73, *n* = 98)	0.0098
	pbp1Δ (16.11, n = 80)	Pbp1-40Q (14.16, *n* = 80)	0.0596
	Pbp1-15Q (14.43, *n* = 79)	Pbp1-30Q (13.73, *n* = 98)	0.5118
	Pbp1-15Q (14.43, *n* = 79)	Pbp1-40Q (14.16, *n* = 80)	0.9825
	Pbp1-30Q (13.73, *n* = 98)	Pbp1-40Q (14.16, *n* = 80)	0.4684
5j	Pbp1-1Q (19.81, *n* = 200)	Pbp1-30Q (13.73, *n* = 98)	< 0.0001
	Pbp1-1Q (19.81, *n* = 200)	*tec1Δ* (17.85, *n* = 40)	0.0942
	Pbp1-1Q (19.81, *n* = 200)	Pbp1-30Q *tec1Δ* (19.38, *n* = 40)	0.6809
	Pbp1-30Q (13.73, *n* = 98)	*tec1Δ* (17.85, *n* = 40)	0.0003
	Pbp1-30Q (13.73, *n* = 98)	Pbp1-30Q *tec1Δ* (19.38, *n* = 40)	< 0.0001
	tec1Δ (17.85, *n* = 40)	Pbp1-30Q *tec1Δ* (19.38, *n* = 40)	0.4294

[a]All strains are isogenic with W303a and mean lifespan followed by the number (*n*) of cells analyzed are listed in parenthesis. Data was pooled from three separate experiments. Only simultaneously counted isogenic strains were compared to each other on graphs and within our statistical analyses.

Supplementary Table 2, related to Fig. 7. Replicative lifespan measurements and *p* values for Mann-Whitney *U* test.

Fig.	Strain A	Strain B	*p* value
7l	Pbp1-1Q (19.81, *n* = 200)	Pbp1-30Q (13.73, *n* = 98)	< 0.0001
	Pbp1-1Q (19.81, *n* = 200)	*fob1Δ* (23.54, *n* = 39)	<0.05
	Pbp1-1Q (19.81, *n* = 200)	Pbp1-30Q *fob1Δ* (25.78, *n* = 39)	< 0.001
	Pbp1-30Q (13.73, *n* = 98)	*fob1Δ* (23.54, *n* = 39)	< 0.0001
	Pbp1-30Q (13.73, *n* = 98)	Pbp1-30Q *fob1Δ* (25.78, *n* = 39)	< 0.0001
	fob1Δ (23.54, *n* = 39)	Pbp1-30Q *fob1Δ* (25.78, *n* = 39)	0.2599

[a]All strains are isogenic with W303a and mean lifespan followed by the number (*n*) of cells analyzed are listed in parenthesis. Data was pooled from three separate experiments. Only simultaneously counted isogenic strains were compared to each other on graphs and within our statistical analyses.
