# Supplementary material for: Conserved Pbp1/Ataxin-2 regulates retrotransposon activity and connects polyglutamine expansion-driven protein aggregation to lifespan-controlling rDNA repeats
Source: Commun Biol. 2018 Nov 5;1:187. doi: 10.1038/s42003-018-0187-3 (PMC6218562; doi:10.1038/s42003-018-0187-3)
Supplement: Supplementary file 3 — Description of Additional Supplementary Files [file 42003_2018_187_MOESM3_ESM.docx]

**Description of Additional Supplementary Files**

**File Name**: Supplementary Data 1

**Description**: This file contains a list of the *Saccharomyces cerevisiae* strains used in this study. For each strain, an identifying strain number and its corresponding genotype is provided.
